# A RAD-sequencing approach to genome-wide marker discovery, genotyping, and phylogenetic inference in a diverse radiation of primates

**DOI:** 10.1371/journal.pone.0201254

**Published:** 2018-08-17

**Authors:** Lina M. Valencia, Amely Martins, Edgardo M. Ortiz, Anthony Di Fiore

**Affiliations:** 1 Primate Molecular Ecology and Evolution Laboratory, Department of Anthropology, University of Texas at Austin, Austin, United States of America; 2 Centro Nacional de Pesquisa de Conservação de Primatas Brasileiros, ICMBio/MMA, Brazil, Brazil; 3 Department of Integrative Biology, University of Texas at Austin, Austin, United States of America; National Cheng Kung University, TAIWAN

## Abstract

Until recently, most phylogenetic and population genetics studies of nonhuman primates have relied on mitochondrial DNA and/or a small number of nuclear DNA markers, which can limit our understanding of primate evolutionary and population history. Here, we describe a cost-effective reduced representation method (ddRAD-seq) for identifying and genotyping large numbers of SNP loci for taxa from across the New World monkeys, a diverse radiation of primates that shared a common ancestor ~20–26 mya. We also estimate, for the first time, the phylogenetic relationships among 15 of the 22 currently-recognized genera of New World monkeys using ddRAD-seq SNP data using both maximum likelihood and quartet-based coalescent methods. Our phylogenetic analyses robustly reconstructed three monophyletic clades corresponding to the three families of extant platyrrhines (Atelidae, Pitheciidae and Cebidae), with Pitheciidae as basal within the radiation. At the genus level, our results conformed well with previous phylogenetic studies and provide additional information relevant to the problematic position of the owl monkey (*Aotus*) within the family Cebidae, suggesting a need for further exploration of incomplete lineage sorting and other explanations for phylogenetic discordance, including introgression. Our study additionally provides one of the first applications of next-generation sequencing methods to the inference of phylogenetic history across an old, diverse radiation of mammals and highlights the broad promise and utility of ddRAD-seq data for molecular primatology.

## Introduction

Molecular genetic studies can provide important and unique insight into the evolutionary history, phylogenetic relationships, migration patterns, and demographic histories of natural populations [1]. Over the past two decades, the field of primatology has benefited greatly from the use of molecular markers to describe and interpret the patterns of genetic variation found within and between primate taxa and to investigate multiple dimensions of primate behavioral biology (e.g., social behavior, kin relationships, dispersal behavior, feeding ecology) and evolutionary history [2–7]. Until recently, however, the discovery of polymorphic markers useful for phylogenetic, phylogeographic, and population genetic studies has been labor-intensive and expensive, and this is particularly true for non-model taxa–like most primates–for which sufficient genomic resources are unavailable [8]. To date, the majority of studies of the evolutionary relationships and population genetics of wild nonhuman primates have relied either on a limited number of sequence based markers (e.g., mtDNA and select nuclear loci) or on short tandem repeat (i.e., STR, or “microsatellite”) loci [9], although that is beginning to change [4,10–15]. These markers are often uninformative when applied outside a narrow set of species of interest, either because homologous loci become increasingly difficult to identify and screen in distantly related taxa or because single-locus measures of genetic divergence become saturated and uninformative at greater time depths. Additionally, despite the fact that some markers, like microsatellites, can display high levels of allelic diversity and thus are very useful for population-level studies–and despite the fact that certain sequence markers are conserved enough to be easily compared among species–it is often the case that relying on small numbers of loci constrains our understanding of the full evolutionary history of a population, given the mosaic nature of genomic evolution [16,17].

With the advent of high-throughput sequencing technologies and their rapidly decreasing costs, it is now possible to study patterns of genetic variation at the genome-wide scale for many taxa of interest, including non-model organisms [18–20]. Still, while the cost of whole-genome sequencing has dropped significantly, sequencing whole genomes of tens or hundreds of individuals–which would be of interest for many phylogeographic and population level studies–remains unfeasibly expensive and is typically unnecessary [21]. Additionally, whole-genome sequencing often generates much more data than needed to answer certain questions of interest, and the practice is still largely limited to model taxa for which reference genomes are available. Because many ecological, functional, population genetic, and phylogenetic questions can be addressed effectively using sequence and/or multilocus genotype data from a more limited set of markers, a number of alternative “next-generation sequencing” (NGS) approaches based on reduced representations of the genome have been developed. These approaches allow researchers to generate large amounts of informative data from many individuals with relatively low cost by systematically targeting only a fraction of the entire genome for sequencing [18,22–25].

Broadly speaking, these approaches can be divided into those that create libraries that are “enriched” for particular genome regions or loci of interest versus those that subsample genomic DNA to yield libraries that comprise an unbiased subset of loci from across the genome [19,24]. Enrichment approaches, such as DNA hybridization capture methods and targeted amplicon sequencing, can be used to select specific coding and/or non-coding regions for a genome of interest (e.g., exons, ultra-conserved element) either for studying neutral genetic variation or for test evolutionary hypotheses. DNA capture methods are often used to increase cost efficiency in population genomics studies using high-quality samples, and they can be used to increase the representation of sequences of interest in libraries generated from noninvasive samples [10,26]. These approaches, however, often rely in the use of a reference genome for the design of capture baits, which makes them less useful for deep phylogenetic studies involving large numbers of non-model taxa [10].

By contrast, “Restriction site-Associated DNA sequencing” (or RAD-seq) is a very powerful and successful method for generating unbiased reduced representation libraries of complete genomes in a relatively easy and inexpensive fashion [18,21–24,27–30]. RAD-seq uses restriction enzymes to digest genomic DNA into numerous DNA fragments without preliminary knowledge of the taxa under study [27,29,31,32]. Digested fragments are then size selected to reduce the number of fragments to be sequenced, and the resulting libraries, in theory, comprise an unbiased subset of loci from across the genome. Sequencing these restriction-site flanked fragments using massively parallel next-generation sequencing platforms allows for the discovery and genotyping of large numbers of polymorphic markers or SNPs in a single step and at lower costs. The lack of reliance on a reference genome and the cost-effectiveness of applying an approach that can be scaled to many individuals makes RAD-seq a promising method to generate comparative genomic data for molecular studies in non-model organisms, like most primate taxa. The ability to screen large numbers of orthologous SNP loci across many individuals at both intra- and interspecific levels also makes this technique extremely useful for addressing questions regarding fine-scale population structure [33], gene flow [34], admixture and hybridization [35], phylogeography [36,37], and phylogenetic relationships [38–41] and can contribute to better precision in population genetic, kinship, and relatedness studies [42,43]. Moreover, if an annotated reference genome is present, specific genes involved in any of the above-mentioned topics can be assayed [44–46], and those regions of the genome responsible for population divergence or responding to natural selection can be pinpointed [23]. Thus far, RAD-seq data have been applied to phylogenetic and evolutionary questions at the species level [36,47–50] and among sets of closely related species [37]. More recent studies have also demonstrated, empirically, the utility of RAD-seq data for inferring phylogenetic relationships among diverse species in clades as old as 23–34 million years [38].

In this study, we assessed the feasibility of using a specific RAD-seq approach known as “double-digest Restriction site-Associated DNA sequencing” (or ddRAD-seq) [51] to discover and genotype thousands of SNPs across taxa spanning the entire radiation of New World monkeys (Infraorder Platyrrhini), a diverse group of primates which comprises three families and up to 22 currently recognized genera, with a last common ancestor dating to 20–26 mya [52–54]. We then demonstrate that these markers can be used for robust phylogenetic inference at multiple taxonomic levels within the platyrrhine radiation.

ddRAD-seq builds on traditional RAD-seq methods by using a combination of two restriction enzymes (typically a common cutter and a rarer cutter) and precise size selection to recover a more tunable number of RAD fragments distributed randomly through the genome. It thus provides greater consistency, uniformity, and replicability across samples in the selection of fragments for sequencing as compared to other methods for generating reduced representation libraries [25]. Because the process is designed to result in reduced representation libraries containing a greater proportion of homologous regions within and among individuals, it also tends to yield higher sequencing depths at each locus, thus helping to ensure that the polymorphisms discovered represent true sequence variants rather than sequencing errors [51].

Because our objective was to develop a protocol (S1 Fig) that was broadly applicable across the entire radiation of New World monkeys, we first tested multiple enzyme pairs and size selection parameters to determine a combination that maximized the consistency of locus recovery across a diverse set of species with different phylogenetic distances to the closest related taxon for which a reference genome is available (common marmosets, *Callithrix jacchus*). We then explored the influence of different assembly pipelines and clustering thresholds on locus recovery and SNP identification. For the former, we tested both a *de novo* clustering algorithm and three other algorithms that map sequence reads to a reference genome. For the latter, we generated data matrices assuming alternative clustering thresholds, within and across samples, for identifying homologous loci. Additionally, using a replicated subset of individuals, we investigated how well our protocol accommodates combinations of data from different independent library preparations.

Finally, to demonstrate the utility of our approach, we used the SNP data generated with our ddRAD-seq protocol to infer the phylogenetic relationships among our samples. Prior molecular studies of New World primate phylogeny have concluded that the three families of extant platyrrhines (Pitheciidae, Atelidae, and Cebidae) diverged rapidly from a common ancestor roughly 20–26 mya [55–61]. Most of these studies have also inferred the same branching pattern among these three families, as well as consistent branching patterns among the different genera of pitheciids and atelids (but see [55,56,61]). Nevertheless, some of these seminal studies have left unresolved a number of important questions about New World monkey evolutionary relationships at lower taxonomic levels, such as the arrangement of the three clades within the Family Cebidae (Aotinae, Callitrichinae, and Cebinae) [55,57,61].

We applied two different methods of phylogenetic inference to our ddRAD-seq data, maximum likelihood and quartet-based species tree inference, and demonstrate that our reconstruction conforms well with prior genetic assessments of the relationships among the three platyrrhine families and among most genera of New World monkeys. Importantly, our results provide additional data that highlight the problematic position of one taxon–the owl monkey (*Aotus*)–within the family Cebidae and suggest a need for further exploration of possible incomplete lineage sorting and/or ancestral gene flow among the cebid subfamilies early in the family’s history. Our study provides one of the first applications of next-generation sequencing methods to the inference of phylogenetic history across a diverse radiation of mammals and highlights the broad promise and utility of ddRAD-seq data for molecular primatology.

## Materials and methods

### Ethics statement

Research permits to collect and export fecal, blood, and tissue samples were provided by the Ecuadorian Ministry of the Environment, the Brazilian Ministry of Environment and the Chico Mendes Institute for Biodiversity Conservation, the Colombian Ministry of Environment and Sustainable Development. Import permits for these samples were provided by the Center for Disease Control and Prevention of the United States of America. IACUC animal care protocols for anesthetization in the field and for tissue/blood sample collection were approved by the University of Texas at Austin (AUP-2014-0248, AUP-2014-00411, AUP-2014-00412, AUP-2016-00044, AUP-2017-00077). Anesthetization involved either darting the animals intramuscularly using PneuDart type P commercial darts in a variety of volume sizes (0.5, 1.0, 1.5, and 2.0 cc) projected from a DanInject CO_2_-powered rifle or by injecting them after live trapping with an appropriate dosage of either Zolatil (tiletmine/zolazepam: 12–18 mg/kg body weight) or ketamine HCl (~25–50 mg/kg body weight), based on published recommendations and estimated body weights [62–65]. Live trapping of individuals was performed using multi-compartment, hand-activated live traps that were baited with ripe bananas following methodologies developed for other Callitrichines [63,66,67]. All protocols were developed and updated in consultation with UT and in country veterinarians and describe in detail the remote anesthetization, examination, health assessment and monitoring, recovery, and sample collection procedures as well as strategies for dealing with risks accompanying these procedures. Trapping and darting procedures were done in the presence of a qualified veterinarian and/or trained project personnel. Samples from captive individuals were donated by the Southwest National Primate Research Center (SNPRC), which is an AAALAC-accredited animal facility, ensuring that it meets the care requirements of both the USDA and the National Research Council Guide for the Care and Use of Laboratory Animals. These requirements ensure adequate space, environmental enrichment, and appropriate consideration of the animals’ social needs. Blood samples used in this study were taken from either sedated animals or from non-sedated animals that are habituated to short-term restraint in a specially designed device that keeps the animal in an upright posture from which the animal can receive a food reward during the process. All blood samples from SNPRC animals were taken under IACUC protocols that are reviewed and approved by the IACUC of the Texas Biomedical Research Institute.

### DNA extraction and quantification

We used DNA isolated from tissue, blood, and hair samples from a total of 53 individuals beonging to 15 of 22 currently-recognized genera of New World monkeys and two genera of Old World monkeys as outgroups (Table 1). These samples represent 20 different species spread across the three currently recognized families of platyrrhines (Cebidae, Atelidae, Pitheciidae). Tissue samples were collected from the margins of both ears using a small punch biopsy (3 to 4mm diameter), while blood samples (up to 40uL) were collected from the femoral artery in heparinized microhematocrit tubes, and several pinches of hair were collected from the base of the tail. For most individuals, fresh genomic DNA was extracted from tissue or blood using the Qiagen DNeasy Blood & Tissue kit (Qiagen) and from hair samples using the Qiagen Forensic DNA MiniKit. For a handful of individuals, we used genomic DNA that had either previously been extracted from samples in the UT Austin collection or provided by colleagues. We quantified the DNA concentration of all samples using the Quant-iT PicoGreen dsDNA Assay Kit (ThermoFisher). Most samples (apart from hair extractions) yielded sufficient genomic DNA for normalization to ~10 ng/ul before digestion and subsequent library construction, with extractions from blood samples having, on average, higher initial DNA concentrations (70.0 ng/ul) than extractions from tissue (36.2 ng/ul) or hair (4.7 ng/ul).

**Table 1:** Samples used in the study to 1) find the optimal restriction enzyme combination, 2) evaluate the effect of sample type on loci recovery, 3) create alternative genotype matrices and reconstruct phylogenetic relationships, and 4) investigate the replicability of the protocol used in this study. Samples used in the phylogenetic analyses are marked with an asterisk (*). All samples from UT Austin were collected in the field for the purpose of this study, while the rest of the samples were donated by colleagues or obtained from existing collections from either captive or field populations.

Species	Family	Sample Code	Sample Type	Country of Origin	Collection	Source
Alouatta seniculus*	ATELIDAE	ASE01	Tissue	Ecuador	UT Austin	Field
Ateles belzebuth*	ATELIDAE	ABE01	Tissue	Ecuador	UT Austin	Field
Ateles belzebuth*	ATELIDAE	ABE02	Tissue	Ecuador	UT Austin	Field
Ateles belzebuth*	ATELIDAE	ABE03	Tissue	Ecuador	UT Austin	Field
Ateles paniscus*	ATELIDAE	APA01	Tissue	Brazil	The Primate Palette: The Evolution of Primate Coloration CPB/ICMBio	Field
Brachyteles arachnoides*	ATELIDAE	BAR01	Extracted DNA	Brazil	CPRJ	Field
Brachyteles hypoxanthus*	ATELIDAE	BHY02	Extracted DNA	Brazil	CPRJ	Field
Lagothrix lagotricha*	ATELIDAE	LLA01	Tissue	Ecuador	UT Austin	Field
Lagothrix lagotricha*	ATELIDAE	LLA02	Tissue	Ecuador	UT Austin	Field
Cacajao melanocephalus*	PITHECIIDAE	CME01	Blood	Captive	UT Austin	Field
Plecturocebus discolor*	PITHECIIDAE	PDIS01	Tissue	Ecuador	UT Austin	Field
Plecturocebus donacophilus*	PITHECIIDAE	PD001	Tissue	Captive	WCS-BZP	Captive
Callicebus barbarabrownae*	PITHECIIDAE	CB001	Tissue	Brazil	MZUSP	Field
Pithecia aequatorialis*	PITHECIIDAE	PAE01	Tissue	Ecuador	UT Austin	Field
Pithecia aequatorialis*	PITHECIIDAE	PAE02	Tissue	Ecuador	UT Austin	Field
Callithrix jacchus*	CEBIDAE	CJA01	Tissue	Captive	SNPRC	Captive
Callithrix jacchus*	CEBIDAE	CJA02	Tissue	Captive	SNPRC	Captive
Cebus albifrons*	CEBIDAE	CAL01	Tissue	Ecuador	UT Austin	Field
Cebus albifrons*	CEBIDAE	CAL02	Tissue	Ecuador	UT Austin	Field
Leontopithecus rosalia*	CEBIDAE	LRO01	Hair	Brazil	SI-NZP	Captive
Saguinus leucopus*	CEBIDAE	SLE01	Tissue	Colombia	UT Austin	Field
Saguinus leucopus*	CEBIDAE	SLE02	Tissue	Colombia	UT Austin	Field
*Saguinus leucopus*	CEBIDAE	SLE03	Tissue	Colombia	UT Austin	Field
*Saguinus leucopus*	CEBIDAE	SLE04	Tissue	Colombia	UT Austin	Field
*Saguinus leucopus*	CEBIDAE	SLE05	Tissue	Colombia	UT Austin	Field
*Saguinus leucopus*	CEBIDAE	SLE06	Tissue	Colombia	UT Austin	Field
*Saguinus leucopus*	CEBIDAE	SLE07	Tissue	Colombia	UT Austin	Field
*Saguinus leucopus*	CEBIDAE	SLE08	Tissue	Colombia	UT Austin	Field
*Saguinus leucopus*	CEBIDAE	SLE09	Tissue	Colombia	UT Austin	Field
*Saguinus leucopus*	CEBIDAE	SLE10	Tissue	Colombia	UT Austin	Field
*Saguinus leucopus*	CEBIDAE	SLE11	Hair	Colombia	UT Austin	Field
*Saguinus leucopus*	CEBIDAE	SLE12	Hair	Colombia	UT Austin	Field
*Saguinus leucopus*	CEBIDAE	SLE13	Hair	Colombia	UT Austin	Field
*Saguinus leucopus*	CEBIDAE	SLE14	Hair	Colombia	UT Austin	Field
*Saguinus leucopus*	CEBIDAE	SLE15	Hair	Colombia	UT Austin	Field
*Saguinus leucopus*	CEBIDAE	SLE16	Hair	Colombia	UT Austin	Field
*Saguinus leucopus*	CEBIDAE	SLE17	Hair	Colombia	UT Austin	Field
Saimiri macrodon*	CEBIDAE	SMA01	Tissue	Ecuador	UT Austin	Field
Sapajus flavius*	CEBIDAE	SFL01	Blood	Brazil	CPB/ICMBio	Field
Sapajus flavius*	CEBIDAE	SFL02	Blood	Brazil	CPB/ICMBio	Field
Sapajus libidinosus*	CEBIDAE	SLI03	Blood	Brazil	CPB/ICMBio	Field
Sapajus libidinosus*	CEBIDAE	SLI02	Blood	Brazil	CPB/ICMBio	Field
*Sapajus libidinosus*	CEBIDAE	SLI03	Blood	Brazil	CPB/ICMBio	Field
*Sapajus libidinosus*	CEBIDAE	SLI04	Blood	Brazil	CPB/ICMBio	Field
*Sapajus libidinosus*	CEBIDAE	SLI05	Tissue	Brazil	CPB/ICMBio	Field
*Sapajus libidinosus*	CEBIDAE	SLI06	Tissue	Brazil	CPB/ICMBio	Field
*Sapajus* sp. indet.	CEBIDAE	SSP01	Tissue	Brazil	CPB/ICMBio	Field
*Sapajus* sp. indet.	CEBIDAE	SSP02	Blood	Brazil	CPB/ICMBio	Field
Sapajus xanthosternos*	CEBIDAE	SXA01	Blood	Brazil	CPB/ICMBio	Field
Sapajus xanthosternos*	CEBIDAE	SXA02	Blood	Brazil	CPB/ICMBio	Field
Aotus vociferans*	CEBIDAE	AOT01	Tissue	Ecuador	UT Austin	Field
*Cercopithecus* sp. (Outgroup)*	CERCOPITHECIDAE	CSP01	Extracted DNA		NYU	Field
*Papio anubis* (Outgroup)*	CERCOPITHECIDAE	PAN01	Extracted DNA		NYU	Field
**TOTAL**					**53 samples**	

UT Austin: University of Texas at Austin

CPRJ: Centro de Primatologia do Rio de Janeiro

CPB/ICMBio: Centro Nacional de Pesquisa e Conservação de Primatas

WCS-BZP: Wildlife Conservation Society–Bronx Zoological Park

MZUSP: Museu de Zoologia da USP

SI-NZP: Smithsonian Institution–National Zoological Park

SNPRC: Southwest National Primate Research Center

NYU: New York University

### Enzyme digestion

To estimate the number of sequenceable RAD fragments (or “RAD tags”) expected using a ddRAD approach, we tested four restriction enzyme pair combinations in a subset of eight of our 53 samples representing four species from four genera in the family Cebidae and three species from three genera in the family Pitheciidae. These enzyme combinations were chosen as they had previously been tested and shown to be effective for generating ddRAD-seq data across a wide range of taxonomic groups, from flowering plants to insects to fish to birds to mammals [51].

**Fig 1:**
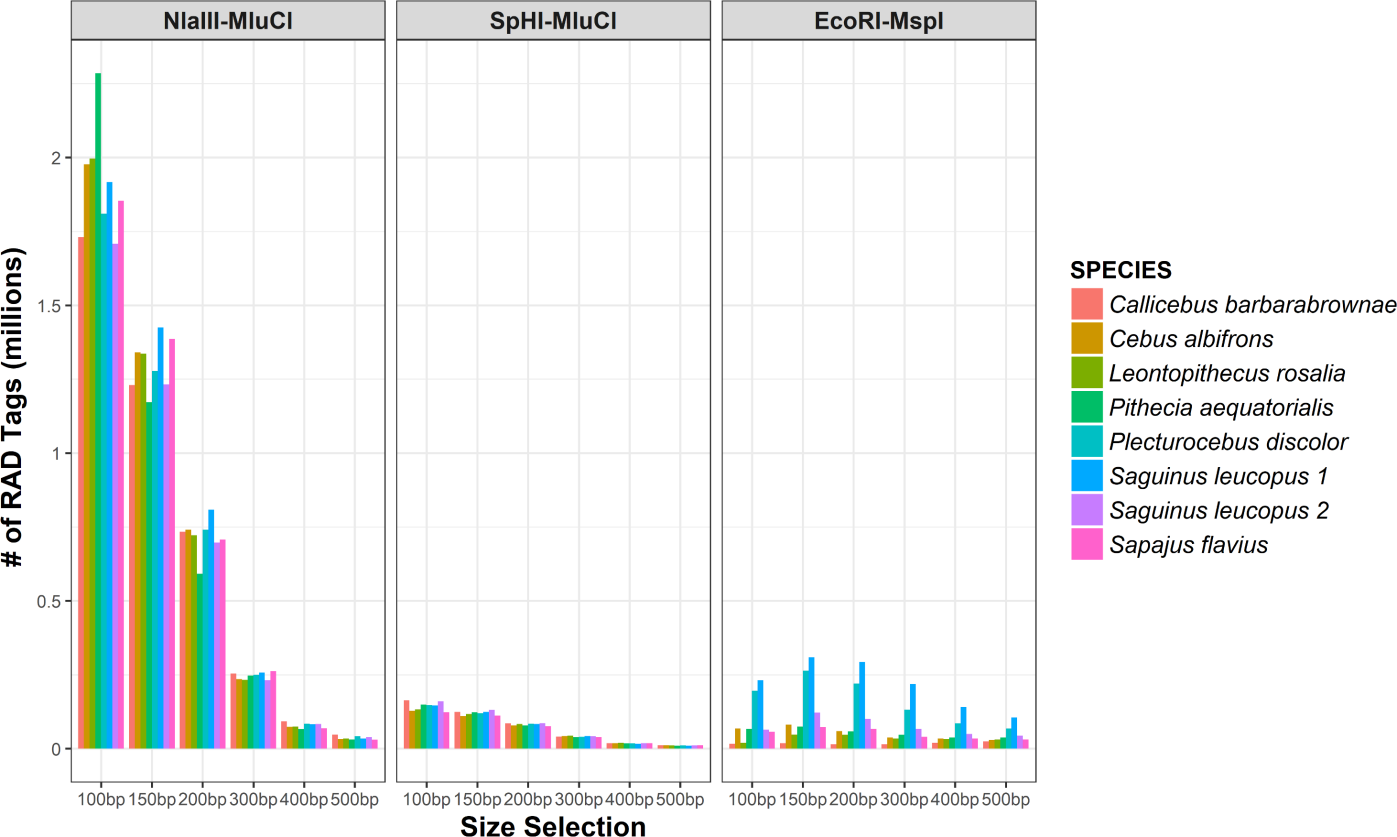
RAD tags recovered for each enzyme pair under different size selections. Note that the SpHI-MluCI and Nlalll-MluCI enzyme combinations yielded relatively even fragment recovery across taxa at each size selection, while the *EcoRI-MspI* enzyme combination was associated with high variation between taxa in the number of RAD tags recovered for each size selection. Also note that, for any given size selection, much more sequencing would be required to yield comparable coverage per fragment for accurately genotyping loci resulting from digestion with Nlalll-MluCI than digestion with SphI-MluCI, thus our choice of the latter combination for this study. See also S1 Table for the actual number of RAD tags recovered in each case.

After normalization, a total of 100ng of genomic DNA for each sample was double digested with the enzyme combinations EcoRI-MspI, SphI-EcoRI, SphI-MluCI, and NlaIII-MluCI. Using a BioAnalyzer, we counted the number of fragments generated when using each of the enzyme pairs under different size selection regimes (i.e., 100, 200, 300, 400, and 500 ± 30 bp). Given that we did not perform single digests of the genomic DNA with each enzyme, we also simulated fragment recovery for digestion with each of the enzymes individually under these different size selection parameters using the R package *simRAD* [68] with the *Callithrix jacchus* genome as a reference (Ensembl version 88—GCA_000004665.1) [69]. We then used the simulated fragment recovery for single digests, in combination with the empirical distribution of fragment sizes resulting from our double-digests, to estimate the number of sequenceable fragments we could expect from each enzyme combination under the alternative size selection parameters. After evaluating the efficiency of each enzyme pair (see Results: Fig 1 and S1 Table), we decided to build ddRAD-seq libraries for our samples using the enzyme pair SphI-MluCI and a fragment size selection of 300 ± 30 bp. With this size selection window, we estimated that we could generate sufficient coverage (≥ 6x) for a genotyping set of ~100,000 RAD tags or loci by targeting a total of only 2–4 million reads per sample, which makes the process very cost effective even for population-level studies.

### ddRAD-seq library preparation and sequencing

We submitted a total of 100 ng of high quality genomic DNA per individual to the Genomic Sequencing and Analysis Facility (GSAF) at the University of Texas at Austin for library preparation and sequencing (see protocol in Supporting Materials S1 File). Briefly, after size selection, P5 and P7 adaptors were ligated to the DNA fragments for each individual sample, and a unique 5 bp sequence tag was added for multiplexing with other samples. To investigate the replicability of our protocol and to evaluate whether the inclusion of replicates from different libraries influenced either the assessment of locus-sharing among individuals or of our phylogenetic inferences, we included replicates for four of our individual samples from three different NWM families in multiple libraries. Our samples were included in a total of seven different libraries prepared at the GSAF and were sequenced in a total ten lanes on an Illumina HiSeq 2500 and two lanes on an Illumina HiSeq 4000 to generate ~150 bp paired-end reads.

### Quality control

Raw sequencing reads were quality checked prior to processing using FASTQC [70] and then filtered using BBDuk.sh from the BBTools package version 34.41 (https://jgi.doe.gov/data-and-tools/bbtools/) [71]. We first trimmed any adapter sequence still present at the 3’ end of the reads using a *kmer* length of 22, allowing a maximum of 3 mismatches and discarding any reads smaller than 30 bp. We specified the “tbo” and “tpe” options to trim adapters based on pair overlap detection as well as to trim all reads to the same length in case an adapter sequence was only detected in one read of a pair. Additionally, we filtered out all reads that mapped to the PhiX genome, as PhiX DNA was used as a spike-in control during library preparation for Illumina sequencing. We verified the correct pairing of R1 and R2 reads and discarded all unpaired reads (“orphans”) from further analysis. Lastly, given the fact that read quality often decreased at the end of a read, we trimmed reads from the terminal end back to the first base that had an average quality score of Q<30.

Sequence reads were then assigned to individual samples (“demultiplexed”) based on their barcode using the program deML [72], allowing for up to one mismatch in the barcode sequence. The resulting set of trimmed and assigned reads thus consisted of, at most, 145 bp beginning with either the 4 bp MluCI or the 6 bp of SphI restriction enzyme recognition sites for the forward (R1) and reverse (R2) reads, respectively.

We further trimmed the demultiplexed sets of reads per individual using the Cutadapt software [73], which removed any remaining P5 and P7 adapter sequences from the 5’ end of each read as well as an additional 5 or 4 bases corresponding to the restriction enzyme recognition sites for the R1 and R2 reads respectively. Additionally, we used Cutadapt to replace all bases with a Phred quality score of less than 20 in each read with Ns. Reads that had more than 5% Ns were then discarded from the dataset. Lastly, we used VSEARCH [74] to assemble corresponding R1 and R2 reads into a single, longer sequence, with a minimum length of 30 bp for the entire merged sequence and a minimum of 20 bp for the length of overlap between the reads and allowing a maximum of four mismatched bases in the overlap region. Both merged and unmerged paired reads were used in our subsequent analyses.

### De novo pipeline for locus identification and SNP calling

The demultiplexed, trimmed, and filtered reads were then used as input for the software iPYRAD v.0.6.24 [39,75] to identify orthologous RAD sequences *de novo*. iPYRAD is unique among the alternative methods for analyzing RAD-seq data because it uses an alignment-clustering method that allows for the inclusion of indel variation, which improves the identification of homology across highly divergent samples. In brief, iPYRAD groups highly similar sequences from the same individual sample into “within sample” clusters. After clustering, iPYRAD jointly estimates the rates of heterozygosity and sequence error across the set of clustered reads within each individual and then, using this information, summarizes each cluster into a consensus sequence. These consensus sequences represent the set of putative loci identified for a particular sample. Loci are then compared and clustered by similarity across individuals to generate a larger matrix of orthologous loci present in the samples being analyzed for downstream analyses. ddRAD-seq data typically does not yield a sequence for every locus in every individual due to mutations in restriction sites in some taxa and/or low sequence coverage at some loci within some individuals. Thus, the resulting matrix of samples by recovered loci is expected to have missing data–i.e., some loci may be present and recovered in most samples, while some may be present or recovered in only a handful.

The key parameter for identifying orthologous RAD sequences within and across individuals is the clustering threshold, which is the level of sequence similarity at which two sequences are identified as being homologous and grouped as a single locus. Setting this threshold too high may split a single locus with divergent alleles, while setting this threshold too low risks grouping non-homologous sequences into a single locus [40]. To evaluate the effect of sequence similarity on the number of loci recovered, we explored different thresholds (ranging from 85% to 95%) for clustering sequences both within and across samples. We did not use thresholds greater than 95% as with such stringent criteria, even truly homologous sequences often may not cluster together due to the presence of uncalled bases (Ns, indels, sequencing errors, or polymorphisms) [76].

Within iPYRAD, reads for each sample were first clustered using VSEARCH [74], and then the sequences within each cluster were aligned using Muscle [77]. To generate a set of loci for each sample, only those clusters with a sequencing depth of at least six reads (≥6x) [78,79] and less than a specified maximum number of heterozygous sites (Hs) and Ns within the consensus sequence were retained (see below). We set the maximum number of Hs and Ns allowed as the upper bound of the 95% CI of these two variables found across the set of consensus sequences (S2 Fig).

Muscle [77] was then used again to align loci across samples to generate a data matrix that included only those loci that were recovered in a minimum of four individuals. We then applied several filters to this data matrix to generate the most complete dataset with no ambiguous genotypes for each sample. First, in order to avoid including potential paralogs, we discarded putative loci containing more than two unique alleles per individual genotype, after accounting for sequencing errors. Second, we filtered out putative loci that were heterozygous in more than 50% of individual samples, as shared heterozygous loci present across many individuals potentially reflect clustering of paralogous sequences rather than true heterozygous sites. Finally, we removed potential effects of poor alignments in repetitive regions by filtering the data matrix to exclude putative loci containing more than a specified maximum number of SNP sites across the entire set of samples. We set the threshold for this maximum number of SNPs as the upper bound of the 95% CI for the distribution of the number of SNPs per locus across all loci (S3 Fig). This process thus yielded a final genotype matrix that we used for subsequent analyses.

### Reference genome-based pipelines for locus identification and SNP calling

One of the species analyzed in this study, *Callithix jacchus*, has a reference genome available. Consequently, as alternative approaches to *de novo* locus identification, we used the *reference* and *denovo+reference* pipelines implemented in iPYRAD [39,75] to identify SNP loci by mapping our ddRAD-seq reads onto the *C*. *jacchus* genome. We then compared how these two reference-based methods performed relative to the *denovo* pipeline described above and evaluated whether the usage of a reference genome could potentially complement *de novo* locus identification.

For both the *reference* and *denovo+reference* pipelines in iPYRAD, we mapped the set of all sequence reads to the *C*. *jacchus* reference genome using the BWA-MEM algorithm from the BWA software package [80]. Additionally, for the *denovo+reference* pipeline, reads that did not align to the reference genome initially were subsequently clustered *de novo* using the method described in the previous section. Locus and SNP identification, and locus filtering for both of the reference-based pipelines were performed as described above for the *denovo* pipeline to yield final genotype matrices for each of these pipelines.

### Phylogenetic analyses

To assess the utility of the loci we recovered using our ddRAD-seq protocol for downstream phylogenetic analyses, we used data from a subset of 33 of our samples. These represented a total of 15 genera and 20 species from across the three platyrrhine families, excluding replicate samples of the same individuals. All of the final genotype matrices used for phylogenetic analyses, as well as the raw reads for each sample used in this study, have been deposited in Dryad (https://doi.org/10.5061/dryad.85jn3).

We used two different approaches to infer the phylogenetic relationships among these samples: 1) a Maximum Likelihood (ML) analysis using the concatenated RAD sequence data from all loci in the final genotype matrix [81] and 2) a coalescent-based approach using quartet-based phylogenetic inference under a multispecies coalescent theory framework [82–84] that also used the concatenated RAD sequence data described above, but only after randomly sampling one SNP per locus. We also explored the effect of using different SNP discovery pipelines (i.e., *denovo* versus *denovo+reference* versus *reference*) on the inference of platyrrhine phylogeny by repeating all of our analyses using the somewhat different sets of loci identified by these alternative methods. We decided to use a quartet-based phylogenetic inference method in addition to ML analysis as an advantage of the former approach is its demonstrated ability to handle large amounts of missing data, as can be common with ddRAD-seq datasets [85]. Moreover, simulation studies comparing quartet-based analysis to other coalescent-based methods, as well as to concatenated sequence dataset analyzed using ML, suggest that the quartet method provides similar results, especially when the amount of incomplete lineage sorting is low and there are few variable sites per locus [86].

We conducted our ML analyses using the IQ-TREE software [87]. The best model of nucleotide substitution and across-site heterogeneity in evolutionary rates was inferred using ModelFinder [88], based on the corrected Akaike’s information criterion. Node and branch supports were obtained from 1000 nonparametric bootstrap replicates [89] under the best inferred model (GTR) [90]. To evaluate significant topological differences between phylogenetic reconstructions obtained from loci identified via the alternate SNP discovery pipelines, we computed the log-likelihood for each competing phylogenetic hypothesis and conducted topology tests using the RELL approximation [91] as implemented in the IQtree software [87]. The tests included comparsion of bootstrap proportions (BP), the Kishino-Hasegawa test [92], the Shimodaira-Hasegawa test [93], a comparison of expected likelihood weights [94], and the approximately unbiased (AU) test [95].

We conducted our quartet-based coalescent phylogenetic inferences using the program Tetrad in the iPYRAD software [39,75,84]. Tetrad implements the SVDquartets algorithm [96], which uses multi-locus unlinked SNP data to infer the topology among all possible subsets of four samples under a coalescent model and then combines the set of resultant quartet trees into a species tree [84,96]. SVDquartets assumes that each SNP site is unlinked and characterized by its own gene tree and, therefore, that each gene tree is independent of the species tree [96]. Thus, in order to guarantee the presence of unlinked SNPs in the data set, for each sampled quartet in each bootstrap replicate, Tetrad randomly sampled a single SNP from the four-taxon alignment at each locus for which they share data. Node supports were again assigned by running 1000 bootstraps.

### Assessment of replicability

To investigate the replicability of our protocol and the feasibility of combining data across different library preparations, we evaluated the influence of technical replicates on locus recovery and phylogenetic topology. Thus, for each of four individuals belonging to the three New World monkey families–two pitheciids, one atelid, and one cebid–we constructed and sequenced three replicate ddRAD libraries using the methods described above. We calculated the percentage of identified loci shared among replicates of a sample as the number of common loci recovered in all three replicates divided by the total number of loci recovered for that sample. We also evaluated the relative positions of all replicates of a sample in our phylogenetic reconstructions.

Finally, we evaluated how increasing genetic divergence between clades impacts the detection of homologous loci across taxa. To do this, we examined the correlation between the number of loci shared among all of the samples within each clade of New World monkeys for which we could extract an estimate of divergence time from already published molecular phylogenies. We also used the R package RADami [38] to generate a pairwise similarity matrix among individuals based on locus sharing.

## Results

### Enzyme combination and size selection

All of the enzyme pairs we tested yielded between thousands and millions of fragments, with the exception of SphI-EcoRI, which produced very few sequenceable fragments in any of the taxa, regardless of what fragment size window was being targeted (S1 Table). Digesting genomic DNA with the enzyme combination EcoRI-MspI produced inconsistent numbers of fragments across taxa, with some species yielding ~20x more fragments than others. By contrast, the number of fragments produced by digestion with the enzyme pairs SphI-MluCI and NlaIII-MluCI was relatively consistent across taxa for all size selections used (Fig 1). For both of these enzyme pairs, as the size of fragments targeted for selection increased, the number of fragments recovered decreased, as expected (Fig 1). Digestion with NlaIII-MluCI yielded significantly more fragments of each of the six different target size windows than digestions with SphI-MluCI (paired T-test: N = 8 samples, p<0.05 for each of six size selections). Although digestion with both of these enzyme pairs resulted in a high and even number of fragments within each size selection across the set of primate taxa being tested, we chose to build our libraries for sequencing with the SphI-MluCI pair because fewer total reads would be needed to achieve the desired sequencing depth (≥ 6x) per locus for accurate genotyping.

We decided to use a size selection window of 300 ± 30 bp for various reasons. First, because the Illumina HiSeq 2500 and 4000 are able to sequence up to 150 bp from each side of a genomic fragment, fragments smaller than ~300 bp are expected to be oversequenced, so targeting smaller fragments would result in less sequencing cost effectiveness. Paired end sequencing of RAD fragments larger than 300 bp, on the other hand, will often yield unmerged reads with gaps in the middle, making alignment and mapping more difficult. Finally, using a wider size selection windows (i.e., more than ± 30 bp) would result in libraries with a heterogeneous set of fragment sizes in which the smaller ones would tend to be over amplified given PCR bias. Thus, we chose to use a narrow size window to provide more consistent library recovery and require less sequencing effort.

### RAD sequences

**Fig 2:**
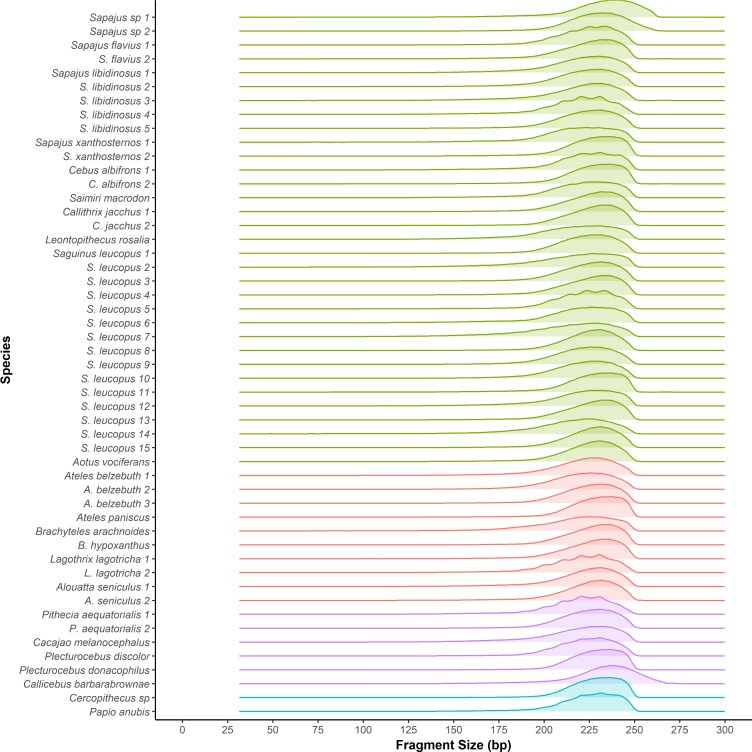
Fragment size distribution. Distribution of fragment sizes of overlapping (merged) reads for each sample used in the study. The average read size for non-overlapping (unmerged) reads was 268 bp.

We generated a total of ~1.9 billion reads in seven libraries across our set of samples, of which ~0.5% and 1.2% were removed for adapter and PhiX genome contamination respectively. All libraries showed excellent quality scores, with reads across libraries having mean Phred scores of 40 or above for both R1 and R2 reads. Nonetheless, quality decreased at the end of the R2 reads, with the last 5 bp having a mean Phred score of 32. Approximately 99% of the reads demultiplexed successfully, and the number of reads varied across samples, with most yielding between 2 and 4 million reads (Table 2). On average, 92% of the R1 and R2 reads per sample overlapped by at least 20 bp (Table 2), and the average size for these merged reads, after removing adapters and applying all the quality filters, was 220 bp (Fig 2). The combined length of R1 plus R2 sequence for non-overlapping (unmerged) paired reads was 268 bp.

**Table 2:** Number of total reads (R1 + R2) after quality filter and demultiplexing.

Species	Family	Sample Code	Barcode	# of Reads Assigned to Sample after Demultiplexing	# of Reads Passing Quality Filter	% of Reads Passing Quality Filter	# of Reads Overlapped (Merged)	% of Reads Overlapped (Merged)
*Alouatta seniculus*	ATELIDAE	ASE01	GCATG	4328764	4326501	99.95	3974239	91.81
*Alouatta seniculus*	ATELIDAE	ASE02	AGCTA	3631636	3629674	99.95	3338723	91.93
*Ateles belzebuth*	ATELIDAE	ABE01	CGAAT	6252196	6164926	98.60	4433453	70.91
*Ateles belzebuth*	ATELIDAE	ABE02	AATTA	1137160	1136606	99.95	1055039	92.78
*Ateles belzebuth*	ATELIDAE	ABE03	ACGGT	3661379	3659247	99.94	3456830	94.41
*Ateles paniscus*	ATELIDAE	APA04	CGATC	2387133	2385339	99.92	2266332	94.94
*Brachyteles arachnoides*	ATELIDAE	BAR01	AATTA	5889147	5885789	99.94	5598910	95.07
*Brachyteles hypoxanthus*	ATELIDAE	BHY01	ACGGT	4589211	4586722	99.95	4268888	93.02
*Lagothrix lagotricha*	ATELIDAE	LLA01	CGATC	4227373	4224781	99.94	3901372	92.29
*Lagothrix lagotricha*	ATELIDAE	LLA02	CGAAT	3324174	3321768	99.93	3139953	94.46
*Cacajao melanocephalus*	PITHECIIDAE	CME01	AGCTA	4070496	4068178	99.94	3795581	93.25
*Plecturocebus discolor*	PITHECIIDAE	PDI01	AATTA	4507308	4504502	99.94	4211716	93.44
*Plecturocebus donacophilus*	PITHECIIDAE	CDO01	ATTAC	4916705	4913608	99.94	4591885	93.39
*Callicebus barbarabrownae*	PITHECIIDAE	CCO1	ATTAC	346306	344871	99.59	313625	90.56
*Pithecia aequatorialis*	PITHECIIDAE	PAE1	CGAAT	3928309	3926414	99.95	3607774	91.84
*Pithecia aequatorialis*	PITHECIIDAE	PAE02	CGAAT	3311659	3309518	99.94	3099272	93.59
*Callithrix jacchus*	CEBIDAE	CJA01	GCATG	4444294	4441554	99.94	4155401	93.50
*Callithrix jacchus*	CEBIDAE	CJA02	AGCTA	1460330	1459473	99.94	1364318	93.43
*Cebus albifrons*	CEBIDAE	CAL01	GCATG	4874470	4871716	99.94	4519397	92.72
*Cebus albifrons*	CEBIDAE	CAL02	TCGAT	2757348	2755073	99.92	2613993	94.80
*Leontopithecus rosalia*	CEBIDAE	LRO01	AATTA	2146527	2145194	99.94	2038312	94.96
*Saguinus leucopus*	CEBIDAE	SLE01	ATGAG	4484167	4481587	99.94	4243595	94.64
*Saguinus leucopus*	CEBIDAE	SLE02	ACGGT	2045103	2043863	99.94	1906861	93.24
*Saguinus leucopus*	CEBIDAE	SLE03	ACTGG	270418	270225	99.93	248896	92.04
*Saguinus leucopus*	CEBIDAE	SLE04	TGCAT	3634520	3632497	99.94	3416870	94.01
*Saguinus leucopus*	CEBIDAE	SLE05	CGGTA	4303557	4300717	99.93	4053199	94.18
*Saguinus leucopus*	CEBIDAE	SLE06	ACTGG	824378	823942	99.95	761346	92.35
*Saguinus leucopus*	CEBIDAE	SLE07	CGTAC	4111658	4109020	99.94	3885062	94.49
*Saguinus leucopus*	CEBIDAE	SLE08	CAACC	3612409	3610350	99.94	3385263	93.71
*Saguinus leucopus*	CEBIDAE	SLE09	CGGCT	4131620	4128922	99.93	3867811	93.61
*Saguinus leucopus*	CEBIDAE	SLE10	TCGAT	4960810	4957854	99.94	4665708	94.05
*Saguinus leucopus*	CEBIDAE	SLE11	CGGCT	4077940	4075983	99.95	3711684	91.02
*Saguinus leucopus*	CEBIDAE	SLE12	ACTGG	2482530	2481494	99.96	2255721	90.86
*Saguinus leucopus*	CEBIDAE	SLE13	TGCAT	212931	212579	99.80	190791	89.60
*Saguinus leucopus*	CEBIDAE	SLE14	ACTTC	4379475	4372296	99.80	4029681	92.00
*Saguinus leucopus*	CEBIDAE	SLE15	ACTGG	216241	215943	99.90	215943	99.90
*Saimiri macrodon*	CEBIDAE	SMA01	TCGAT	824378	824017	99.90	701159	85.10
*Sapajus flavius*	CEBIDAE	SFL01	ACACA	3311659	3309873	99.90	3023017	91.30
*Sapajus flavius*	CEBIDAE	SFL02	ACACA	4444294	4441834	99.90	3943070	88.80
*Sapajus libidinosus*	CEBIDAE	SLI01	CATAT	346306	346059	99.90	299030	86.40
*Sapajus libidinosus*	CEBIDAE	SLI02	ACACA	3928309	3926609	99.90	3218517	81.90
*Sapajus libidinosus*	CEBIDAE	SLI03	CATAT	2210461	2207398	99.90	2018354	91.30
*Sapajus libidinosus*	CEBIDAE	SLI04	AACCA	3423115	3418198	99.90	3103413	90.70
*Sapajus libidinosus*	CEBIDAE	SLI05	AACCA	1569130	1566798	99.90	1451588	92.50
*Sapajus libidinosus*	CEBIDAE	SLI06	ACACA	1378251	1376197	99.80	1278844	92.80
*Sapajus* sp. indet.	CEBIDAE	SSP01	CATAT	3135358	2865586	91.40	3131053	99.90
*Sapajus* sp. indet.	CEBIDAE	SSP02	AACCA	117295	104425	89.00	117107	99.80
*Sapajus xanthosternos*	CEBIDAE	SXA01	ATTAC	4507308	4913955	99.90	4002359	81.50
*Sapajus xanthosternos*	CEBIDAE	SXA02	AGCTA	4916705	4857808	99.90	4362318	89.80
*Cercopithecus* sp.	CERCOPITHECIIDAE	CSP01	CTGAT	4960810	4958272	99.90	4447434	89.70
*Papio anubis*	CERCOPITHECIIDAE	PAN01	AATTA	3634520	3632675	99.90	3232055	88.90

Number of total reads (R1 + R2) recovered, per sample, after demultiplexing, number and percentage of reads that passed quality filters, and number and percentage of reads that overlapped (merged) successfully.

**Fig 3:**
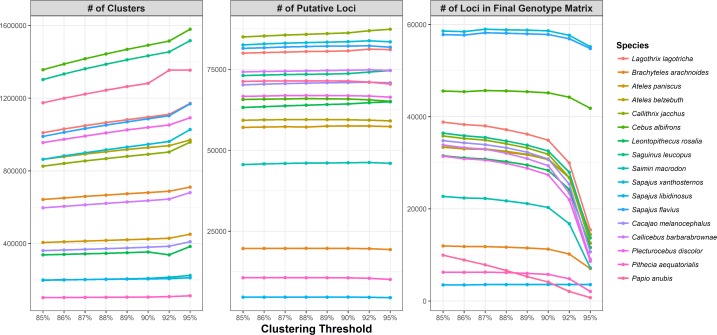
Influence of clustering threshold on the number of clusters recovered per sample, the number of putative loci recovered per sample, and the total number of loci for each sample included in the final genotype matrix. As the clustering threshold increases, the number of clusters and the number of putative loci per sample increases, but the total number of loci per sample in the final genotype matrix decreases.

Not surprisingly, as observed in other studies, as we increased the sequence similarity clustering threshold used to identify clusters, both the number of clusters and the number of putative loci obtained per sample increased slightly, but the number of loci shared across samples (and thus, by extension, the number of total loci per sample in the final genotype matrix) decreased [39, 85]. The most dramatic change in the number of clusters identified was seen at a threshold value of 92% (Fig 3).

The number of reads and the number of putative loci recovered for each sample differed significantly across sample types (hair versus blood versus tissue) [ANOVA: F_2, 46_ = 4.917 and 6.272 for reads and for putative loci, respectively, p<0.05 for both comparisons]. Hair samples yielded significantly fewer reads [Tukey HSD: p<0.05] and significantly fewer putative loci [Tukey HSD: p<0.05] than either blood or tissue samples, which did not yield significantly different numbers from one another (S4 Fig).

When mapping the total set of R1 and R2 reads from each sample to the *Callithrix jacchus* reference genome, an average of 93±1.7% SD aligned successfully. Additionally, for the 92% of paired reads that could be merged (i.e., where R1 and R2 reads overlapped by at least 20 bp to yield a single sequence), 98% mapped successfully to the reference genome. For the remaining 8% of paired reads that were unmerged, 37% nonetheless had R1 and R2 reads that also mapped to the same chromosome within a reasonable distance of one another (± 4 standard deviations from the insert size), and only 1% of unmerged paired reads had only one of their R1 or R2 sequences map successfully to the genome. Thus, a total of fewer than 5.8% of paired reads (0.08 x (1–0.37–0.01)) did not map successfully to the *Callithrix jacchus* genome.

### Comparison of locus identification pipelines

Across the set of samples, the total number of putative loci identified–as well as the number of loci removed in the various filtering steps in iPYRAD–varied from pipeline to pipeline (Table 3). The *denovo* pipeline initially identified the greatest number of putative loci (323,183), while the *reference* pipeline identified the least (274,326). In the *denovo+reference* and *reference* pipelines, more putative loci were discarded as likely paralogs (i.e., either duplicate loci or loci with more than the max number of alleles allowed per locus across samples), while in the *denovo* pipeline more putative loci were discarded based on their exceeding a threshold number of variable SNP sites within the locus (thus likely representing cases of poor sequence alignment) (Table 3). Overall, the number of total loci in the final iPYRAD genotype matrices was highest for the *denovo* pipeline followed by the *denovo+reference* and *reference* pipelines (Table 3, Table 4). For each iPYRAD pipeline, more than 98% of the loci included in the final genotype matrix were also found to be variable across the set of samples examined (Table 4).

**Table 3:** Number of putative loci identified across all samples for each iPYRAD pipeline and number retained after each filtering step.

Pipeline	*denovo*			*denovo+reference*			*reference*		
	# Loci Filtered	% Loci Filtered	# Loci Retained	# Loci Filtered	% Loci Filtered	# Loci Retained	# Loci Filtered	% Loci Filtered	# Loci Retained
**Total # of Putative Loci Across All Samples** **(≥ 6x Coverage)**	–	–	323,183	–	–	316,370	–	–	274,326
**Filtering to Remove Duplicates**	12,676	3.9	310,507	19,871	6.3	296,499	6,116	2.2	268,210
**Filtering Loci by Max # SNPs**	40,817	13.1	269,741	10,493	3.5	287,084	9,446	3.5	259,131
**Filtering Loci Recovered in Fewer Than 4 Samples**	173,053	64.2	97,741	177,187	61.7	115,769	152,335	58.8	109,099
**Filtering by Max # Alleles**	32,810	33.6	86,407	68,832	59.5	77,035	62,635	57.4	71,322
**# Loci in Final Genotype Matrix**	**–**	**–**	**86,407**	**–**	**–**	**77,035**	**–**	**–**	**71,322**

Loci retained after removing potential paralogs (i.e. likely duplicates and loci with more than two alleles per locus per individual), loci that had more than a maximum number of variable sites, and loci that were not recovered in at least 4 individuals. Values in the last row indicate the total number of loci included in the final genotype matrix for each iPYRAD pipeline.

**Table 4:** Total number of loci and number of variable loci in the final genotype matrices, and the proportion of those loci that were variable, based on each of the analysis pipelines.

Pipeline	*denovo*	*denovo+reference*	*reference*
**# Loci in Final Genotype Matrix**	86,407	77,035	71,322
**# of Loci Variable across Samples**	84,834	75,680	70,145
**% of Loci Variable across Samples**	98.2	98.2	98.3
**Total # of SNP sites across Loci**	1,515,545	1,867,289	1,735,513

**Fig 4:**
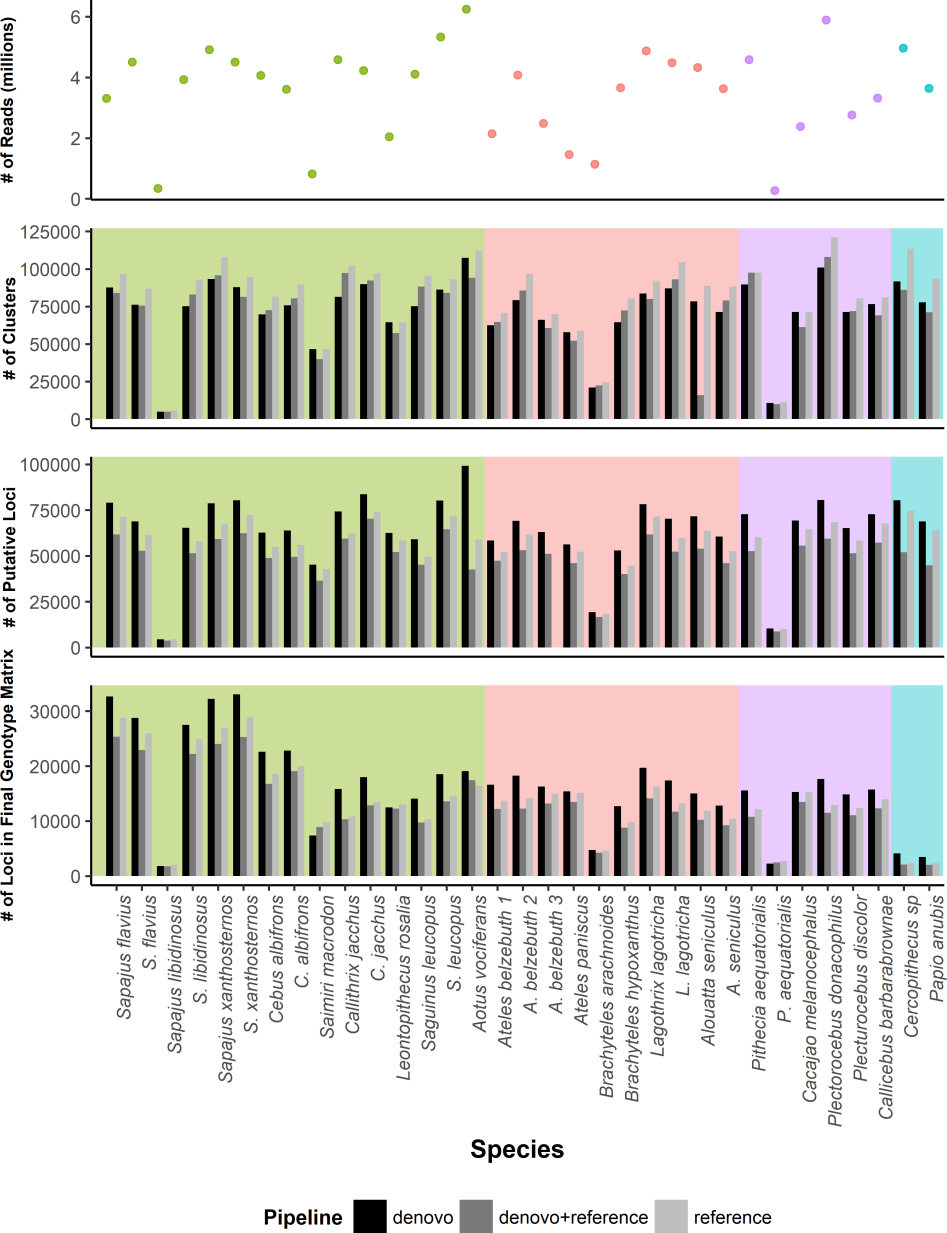
Number of reads, number of clusters, number of putative loci, and number of loci in the final genotype matrix after filtering for each of the three iPYRAD pipelines. Note that the number of clusters and the number of putative loci per sample was highest for the *denovo+reference* pipeline, but the total number of loci per sample in the final genotype matrix was highest for the *denovo* pipeline. In each figure, the three platyrrhine families are indicated by background shading (green: Cebidae, pink: Atelidae, magenta: Pitheciidae. OWM are indicated by teal).

The total number of within-sample clusters recovered and the number of putative loci per sample were both highest for the *denovo+reference* pipeline (Fig 4, Table 5). The *denovo* and *reference* pipelines in iPYRAD each recovered an intermediate number of clusters and putative loci per sample, with no clear pattern across samples as to which of these pipelines identified a greater number (Fig 4, Table 5). However, after all of the filtering steps, the number of loci per sample in the final genotype matrix was highest for the *denovo* pipeline. In addition, for all the three pipelines there was a significant positive relationship between the number of reads and the number of putative loci recovered per sample [Pearson’s R ranged from 0.56 to 0.63, all p<0.01] as well as between the number of reads and the mean per locus sequencing depth [Pearson’s R = 0.70 to 0.72, all p<0.01]. Across samples, the average sequencing depth per recovered locus also did not differ significantly between pipelines [ANOVA: F_2, 96_ = 1.959, p > 0.05].

**Table 5:** Number of putative loci recovered, average sequencing depth, number of loci present in the final genotype matrix, and estimated percentage of the genome recovered for each sample using the three iPYRAD pipelines.

	Pipeline	*denovo*				*denovo+reference*				*reference*			
Family	Species	Total # Loci Recovered [A]	Average Coverage (x) [B]	# Loci in Final Genotype Matrix [C]	Estimated % of Genome Sampled [D[	A	B	C	D	A	B	C	D
**ATELIDAE**	*Alouatta seniculus*	60138	31.8	16895	0.42	76080	30.7	16173	0.50	61504	30.6	14748	0.40
	*Alouatta seniculus*	47380	19.6	14669	0.33	60035	18.8	13860	0.40	50550	18.7	12897	0.40
	*Ateles belzebuth*	47654	14.5	18809	0.34	52918	14.2	16371	0.40	46680	13.9	15247	0.30
	*Ateles belzebuth*	130881	16.5	21046	0.92	130834	15.9	18673	0.90	109183	15.9	17164	0.80
	*Ateles belzebuth*	63326	26.7	18065	0.45	76355	27.3	16394	0.50	62800	27.6	14973	0.40
	*Ateles paniscus*	21856	32.8	16972	0.16	35850	31.0	15791	0.30	30665	31.0	14419	0.20
	*Brachyteles hypoxanthus*	11043	21.4	5197	0.08	15248	20.1	5026	0.10	13334	19.5	4687	0.10
	*Brachyteles arachnoides*	47349	32.2	14434	0.33	50735	32.1	12714	0.40	44945	31.8	11917	0.30
	*Lagothrix lagotricha*	42650	33.5	22300	0.31	64019	32.6	20914	0.50	52560	32.9	19122	0.40
	*Lagothrix lagotricha*	45286	16.4	20157	0.33	57411	16.9	17502	0.40	49506	16.9	16335	0.40
**CEBIDAE**	*Aotus vociferans*	71440	36.8	19072	0.5	85156	42.2	16371	0.60	74567	44.9	17428	0.50
	*Callithrix jacchus*	44837	36.3	17668	0.33	49635	35.9	14674	0.40	45551	36.3	14413	0.30
	*Callithrix jacchus*	48548	29.7	20279	0.36	63064	28.7	18122	0.50	58469	28.9	17737	0.40
	*Cebus albifrons*	41140	11.2	24446	0.26	46120	10.4	21561	0.30	37207	9.8	19990	0.20
	*Cebus albifrons*	38954	25.0	25091	0.26	48410	23.1	22537	0.30	40044	23.0	21029	0.30
	*Leontopithecus rosalia*	27602	41.9	13830	0.21	37201	40.2	14691	0.30	29551	40.5	14109	0.20
	*Saguinus leucopus*	30084	24.4	15975	0.22	34843	22.2	14006	0.30	28995	21.9	13583	0.20
	*Saguinus leucopus*	37497	20.4	20959	0.28	49132	20.1	19484	0.40	40122	20.2	18696	0.30
	*Saimiri macrodon*	20969	12.1	8019	0.16	27767	12.4	9530	0.20	21568	12.5	8958	0.20
	*Sapajus xanthosternos*	46060	20.7	35765	0.32	58440	19.9	32127	0.40	45985	19.9	29096	0.30
	*Sapajus xanthosternos*	3300	32.5	1845	0.02	2327	32.5	1681	0.00	1582	32.7	1455	0.00
	*Sapajus flavius*	31424	31.1	30017	0.22	41947	27.2	27611	0.30	33331	27.0	25380	0.20
	*Sapajus flavius*	54676	27.3	36187	0.38	71202	25.5	32581	0.50	57693	25.5	29412	0.40
	*Sapajus libidinosus*	38426	20.7	31279	0.27	45617	19.9	27994	0.30	34663	19.9	25420	0.20
	*Sapajus libidinosus*	51941	24.3	35283	0.36	67030	24.2	30705	0.50	55395	24.6	28198	0.40
**PITHECIIDAE**	*Cacajao melanocephalus*	30941	31.8	17180	0.34	43105	30.7	18164	0.50	35003	31.0	16532	0.40
	*Plecturocebus discolor*	43889	20.8	17746	0.43	59502	19.4	17651	0.60	48214	19.3	16171	0.50
	*Pithecia aequatorialis*	5719	14.7	2367	0.06	4880	14.8	2436	0.10	4042	14.9	2166	0.00
	*Pithecia aequatorialis*	33572	22.8	18011	0.38	44242	22.0	16545	0.50	36624	22.2	15383	0.40
	*Callicebus barbarabrownae*	38074	24.2	16763	0.42	46492	25.0	16061	0.50	38980	24.4	14952	0.40
	*Plecturocebus* *donacophilus*	48936	9.2	20412	0.55	65014	9.1	18098	0.70	54517	8.7	16777	0.60
**OWM**	*Cercopithecus* sp.	87686	25.0	4752	0.46	93672	24.0	3936	0.50	62860	24.2	3674	0.30
	*Papio anubis*	45248	19.6	4028	0.33	51863	19.3	3610	0.40	34295	19.2	3280	0.20
	**AVERAGE**	**43592**	**24.5**	**18349**	**0.33**	**53217**	**23.9**	**16776**	**0.41**	**43666**	**23.9**	**15617**	**0.32**

OWM: Old World Monkeys (Cercopithecidae)

On average, the *denovo+reference* pipeline yielded a higher total number of putative loci per sample than the other two pipelines, while the *denovo* pipeline identified the greatest number of loci per sample, in the final genotype matrix (and, thus, also yielded the greatest average estimated proportion of the genome sampled). Note that results are only shown for those 33 samples used in our phylogenetic analyses, although the pattern is similar for the remaining samples.

We estimated the percentage of the genome recovered through our reduced representation sequencing by using information about the number of putative loci recovered per individual sample, the average RAD tag size (i.e., 300 bp, based on our size selection), and the estimated total genome size for each genus (www.genomesize.com). The estimated proportion of the genome sequenced varied among genera, from an average of 0.19% in *Brachyteles* to an average of 0.60% in *Cacajao* (Table 5). We recovered a slightly lower estimated proportion of the genome for the families Atelidae (mean = 0.32%, N = 4 genera) and Cebidae (mean = 0.29%, N = 7 genera) compared to the Pitheciidae (mean = 0.46%, N = 4 genera), although this difference was not significant [ANOVA: F_2,12_ = 1.924, p>0.05].

When looking at only the results from the *denovo* pipeline, we recovered a total of 88,266 loci within the set of New World monkey samples, 86,670 (98.4%) of which were variable within this set of taxa (results for the other pipelines are similar). We also recovered tens of thousands of variable loci for each New World monkey family (Atelidae: 41,063, Cebidae: 67,789, Pitheciidae: 22,445). Not surprisingly, as we increased the minimum number of samples that a locus had to be present in for inclusion in the final genotype matrix, both the number of total loci and the number of variable loci identified decreased (S2 Table).

**Fig 5:**
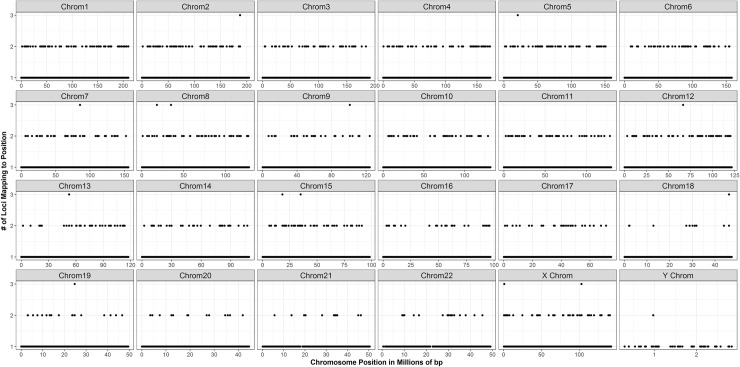
Mapping of loci discovered using the *denovo* pipeline to the *Callithrix jacchus* reference genome. 70% of the loci in the final genotype matrix mapped successfully to the *Callithrix* genome. Only 1% of loci mapped to the same genome locations, indicating that the pipeline successfully filtered out duplicate and paralogous loci. Additionally, loci mapped evenly across and within chromosomes, with no significant gaps (see also S5 Fig).

**Fig 6:**
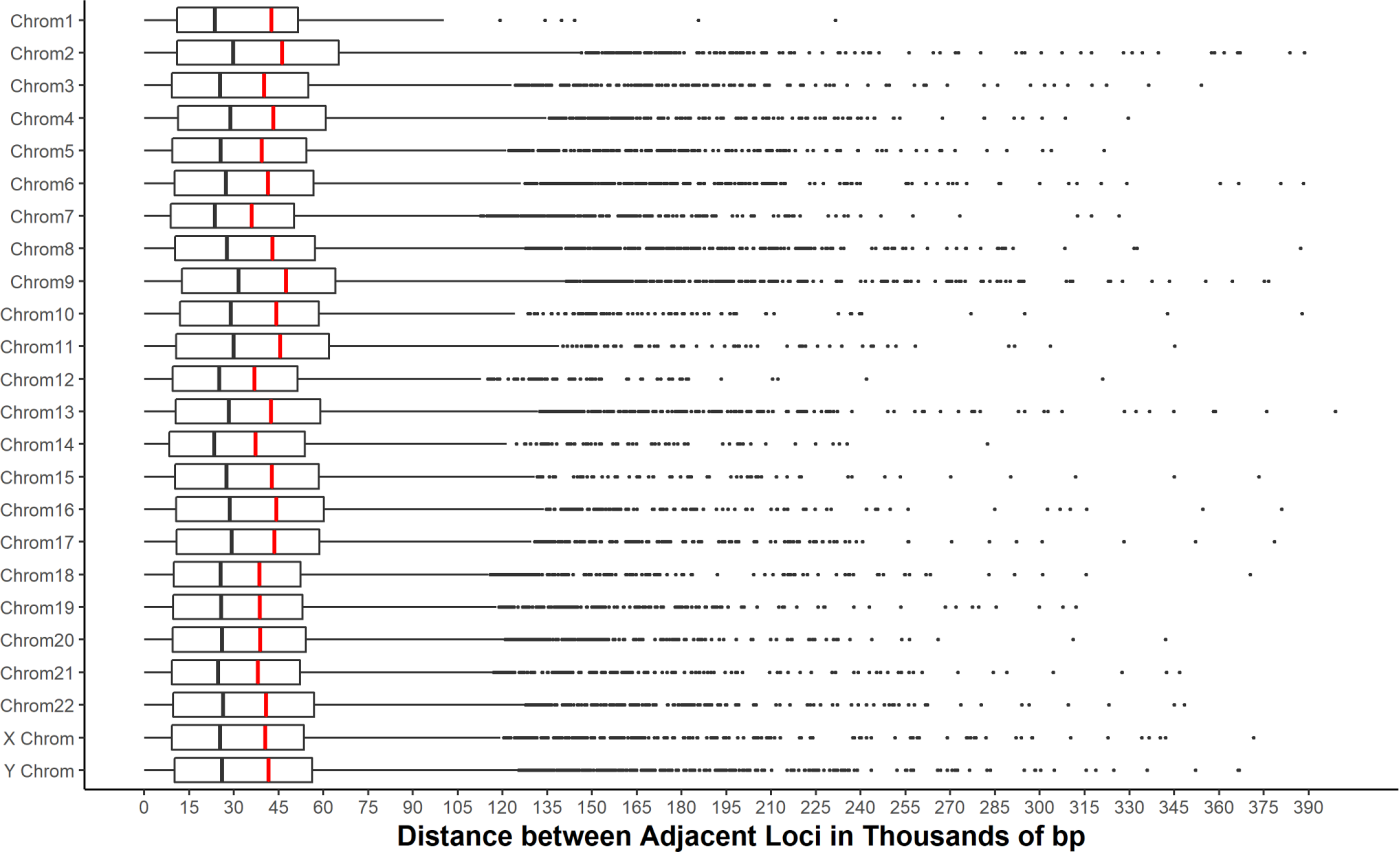
Distribution of distances between adjacent loci recovered using the *denovo* pipeline that map to the *Callithrix jacchus* genome. Red lines indicate the mean distance on each chromosome, black lines the median distance. Across chromosomes, on average, we identified a SNP locus every ~41,751 bp (± 49,312 SD).

Of the 86,407 loci in the final genotype matrix based on the *denovo* pipeline for our whole set of samples (31 New World monkeys + 2 Old World monkey), 70% (N = 59,904 loci) mapped successfully to the *Callithrix jacchus* reference genome, and more than 99% of these loci mapped to a unique location (Fig 5 and S5 Fig). This result suggests that the parameters used in the *denovo* pipeline indeed successfully filtered out most paralogous loci. Fig 5 also shows the distribution of locus recovery across and within chromosomes, demonstrating the ability of the ddRAD-seq approach to identify loci evenly and with no significant gaps across the entire genome. On average, we discovered a locus every ~41,751bp (± 49,312 SD), with a median distance between loci of 29,249 bp (Fig 6).

### Phylogenetic inferences

**Fig 7:**
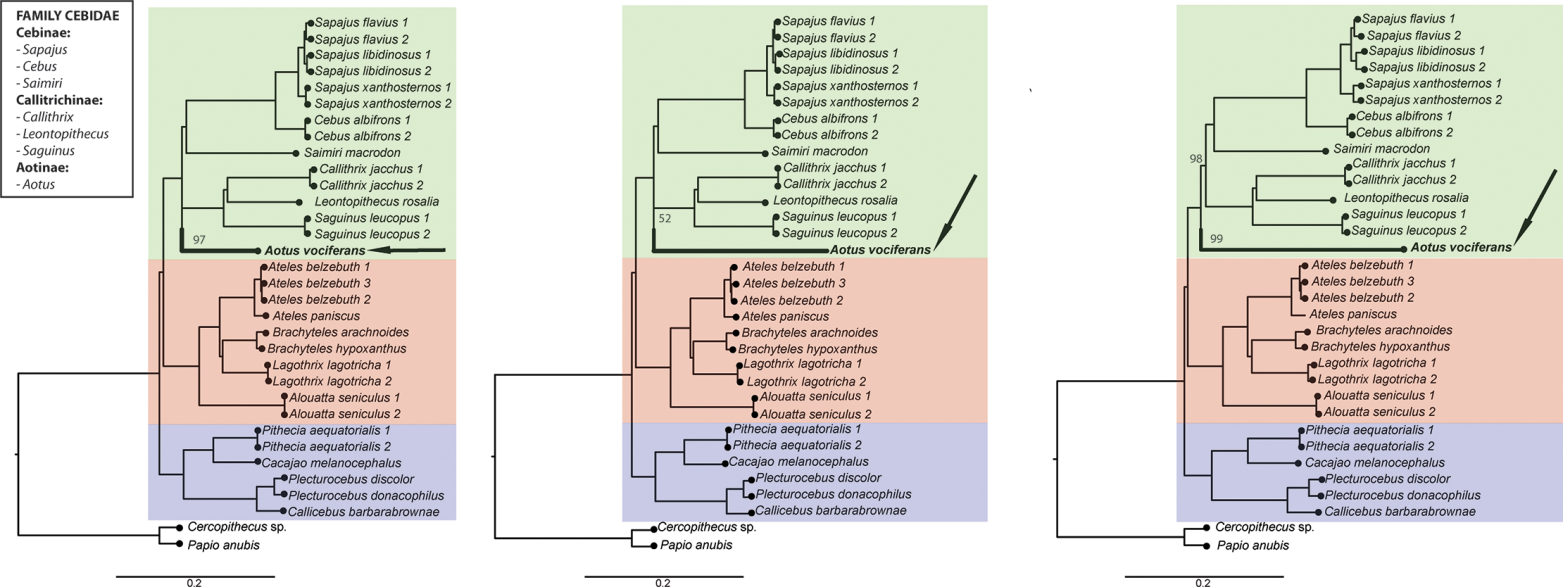
**Phylogenetic relationships among the samples included in our study based on maximum likelihood analyses of loci identified through the (a) *denovo*, (b) *denovo+reference*, and (c) *reference* pipelines in iPYRAD.** Data for each taxon consisted of the concatenated SNPs from all ddRAD loci. In each figure, the three platyrrhine families are indicated by background shading (green: Cebidae, red: Atelidae, blue: Pitheciidae). Numbers in each figure indicate nonparametric bootstrap support for the adjacent node. All unlabeled nodes had 100% bootstrap support. The position of *Aotus* is indicated in bold and by an arrow in each figure.

Our ML phylogenetic analyses using the final genotype matrices resulting from all three iPYRAD pipelines converged on a single tree topology for all but one internal node–the position of the genus *Aotus*–and with all but this one internal node and two other internal nodes within the family Cebidae across the three pipelines showing 100% support in our nonparametric bootstraps (Fig 7). Using the *denovo* final genotype matrix, *Aotus* was reconstructed as the sister taxon to the Callitrichinae (marmosets and tamarins) with 97% bootstrap support (Fig 7A), although with a branch length of close to zero (<0.0000001) between the last common ancestor of all cebids and the last common ancestor of *Aotus*+callitrichines. In the analysis of the *denovo+reference* and *reference* pipeline matrices, however, the inferred position of *Aotus* shifted to being basal within the Cebidae, with 100% and 99% bootstrap support respectively (Fig 7B and 7C), but with minimal branch length between the last common ancestor of all cebids and the last common ancestor of cebines+callitrichines. The very short branch between the cebid common ancestor and either the last common ancestor of *Aotus*+callitrichines (for the *denovo* matrix) or the last common ancestor of cebines+callitrichines (in the two reference-based matrices)–coupled with the low bootstrap support (52%) we found for a clade of cebines+callitrichines using the *denovo+reference* genotype matrix–strongly suggest that the relationships among the three lineages within the Cebidae still cannot be resolved with confidence using even the large set of loci identified under each of the three pipelines. Moreover, topology tests were not able to reject either of the two most likely arrangements recovered in our phylogenetic analyses (i.e., that *Aotus* is sister to the Callitrichinae or that *Aotus* is basal within the Cebidae), and the difference in the likelihoods of the two topologies was not significant for any of the three genotype matrices (*denovo* ΔlnL = 0.001, *denovo+reference* ΔlnL = 0.056, *reference* ΔlnL = 1.633, all NS).

**Fig 8:**
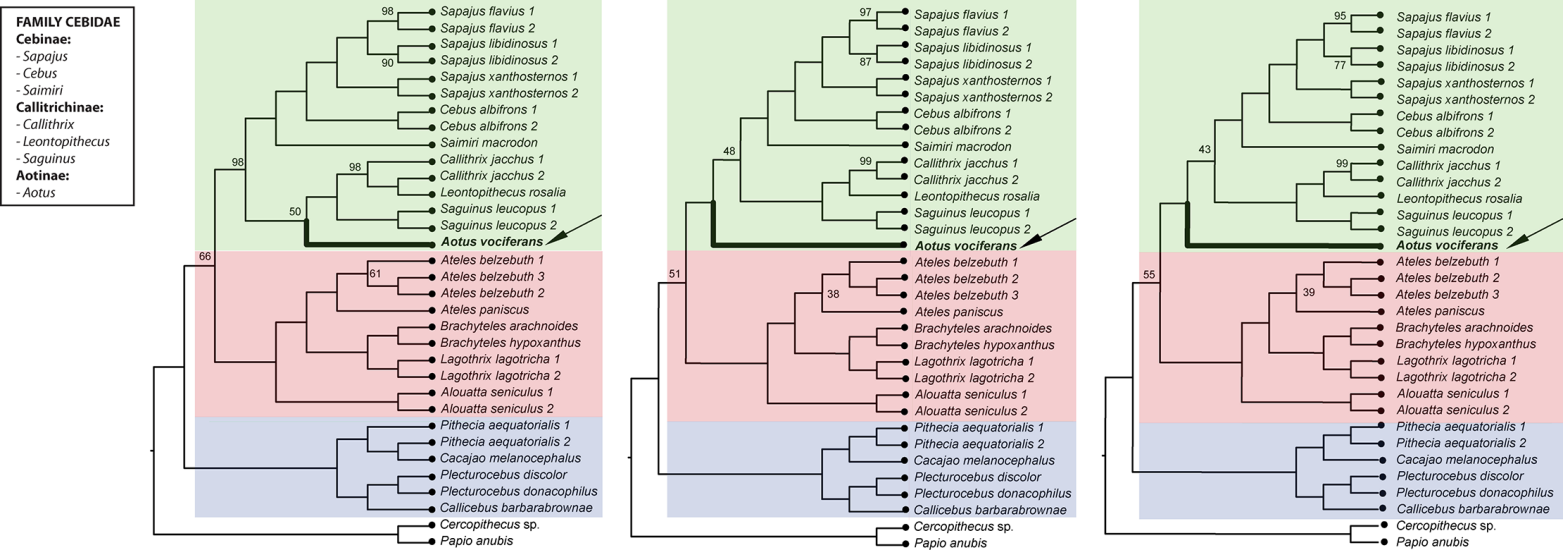
**Phylogenetic relationships among the samples included in our study based on quartet multispecies coalescent analyses of loci identified through the (a) denovo, (b) denovo+reference, and (c) reference pipelines in iPYRAD.** Data for each taxon consisted of a single, randomly chosen SNP site from each ddRAD locus. As in Fig 7, the three platyrrhine families are indicated by background shading (green: Cebidae, red: Atelidae, blue: Pitheciidae). The numbers at each node indicate percent support for the node across 1000 replicate quartet analyses, and all unlabeled nodes had 100% support. The position of *Aotus* is indicated in bold and by an arrow in each figure.

Similarly, our quartet-based coalescent analyses using the final genotype matrices from the *denovo* (77,228 SNPs and 40,920 quartet tree sets), *denovo+reference* (65,685 SNPs and 35,960 quartet tree sets), and *reference* (62,099 SNPs and 40,920 quartet tree sets) pipelines all yielded identical species-level topology to those inferred using ML analysis of data from the same pipelines. In the quartet-based tree using the genotype matrix from the *denovo* pipeline, the position of *Aotus* as sister to the Callitrichinae had only weak bootstrap support (50%) (Fig 8A), while in the trees based on the *denovo+reference* and *reference* matrices, the alternative topology of a sister relationship between the callitrichines and cebines was likewise only weakly supported (48% and 43% for these two data sets, respectively: Fig 8B and 8C). Additionally, in the quartet analyses of all three data sets, the deeper sister relationship between the Atelidae and Cebidae received much weaker bootstrap support than was seen in the ML analyses (66%, 51%, and 55% support for the *denovo*, *denovo+reference*, and *reference pipelines*, respectively, versus 100% support in all of the likelihood analyses).

When *Aotus* is not included in the final data matrices, a sister relationship of Atelidae and Cebidae received 100% bootstrap support in all phylogenetic analyses using both ML quartet methods (S6 and S7 Figs respectively). This result suggests that the observed variable position of *Aotus* within the Cebidae, the very short branches seen in the early radiation of that family (depending on the dataset and type of phylogenetic analysis being performed), and the weaker support for an atelid-cebid sister grouping seen in the quartet analyses may be due to incomplete lineage sorting and/or other evolutionary processes that can create phylogenetic uncertainty (e.g., introgression and ancestral hybridization) among early members of the Cebidae.

Overall, our phylogenetic analyses strongly support monophyly for each of the three currently recognized families of platyrrhines (Pitheciidae, Atelidae, and Cebidae) and a basal position for the Pitheciidae within the platyrrhines. Apart from the position of *Aotus*, all of our analyses affirm previous phylogenetic reconstructions of the arrangement of the genera represented in our samples [52,57,58,60,97]

Across platyrrhines, the number of loci shared between clades decreased as evolutionary divergence time increased, although this relationship was not significant (Pearson’s R: -0.34, -0.42 and -0.44; p > 0.05 for all comparisons to dates presented in [52,53,58], respectively) (S8 Fig). Not surprisingly, however, the pattern of locus sharing across taxa showed evidence of being phylogenetically structured, with more closely related taxa sharing more loci with one another than more distantly related taxa.

### Replicate libraries

The number of sequence reads obtained across replicate libraries of the same sample differed, with some replicates yielding twice the number of reads as their counterparts (S2 Table). However, the proportion of common loci identified across replicates was high with, on average, ~68% of loci discovered being shared across the entire set of replicates for each sample (S3 Table). When replicate samples were included in our phylogenetic analyses, they were invariably reconstructed as sister taxa, with branch lengths of zero separating them in the phylogeny (data not shown). Despite the inherent stochasticity of the ddRAD protocol, the fact that we sequenced a relatively small number of loci per sample (over 44,000, on average) with relatively high coverage (over 20x, on average), enhanced the replicability of the protocol and the robustness of the genotyping techniques employed.

## Discussion

Our study outlines and demonstrates the effectiveness of a general ddRAD-seq protocol for identifying large numbers of variable markers suitable for phylogenetic studies in a diverse group of primates over a range of taxonomic levels and evolutionary time scales. First, we tested different enzyme pairs and provide empirical evidence of which combination performed best at producing comparable reduced representation RAD libraries (in terms of numbers and distributions of fragments of different size) across a wide range of platyrrhine taxa. Second, we generated different genotype matrices using alternative clustering thresholds and locus identification pipelines to evaluate the influence of these parameters on locus discovery. Finally, we identified and genotyped between ~70,000 and ~138,000 variable RAD loci across the whole dataset using three different locus identification pipelines and used the resultant genotype matrices to reconstruct molecular phylogenies for New World monkeys, a clade that diverged from other anthropoid primates ~37 to 43 mya and whose most recent common ancestor dates to ~20 to 26 mya [52,53]. Apart from the position of *Aotus*, these phylogenic reconstructions were all strongly resolved and strongly supported.

### Double enzyme digests

Our initial double-digests revealed that not all enzyme pairs worked consistently well across primate taxa. Only two of the four enzyme combinations (SphI-MluCI and NlaIII-MluCI) yielded comparable numbers of RAD tags across the set of eight species from two platyrrhine families that we tested initially. The combination SphI-MluCI also produced a reasonable number of loci–tens of thousands–that could be consistently sequenced at sufficient depth to identify informative polymorphism without exorbitant sequencing costs. This result provides useful data for new researchers and genomic facilities working on other non-model mammalian taxa. The general ddRAD-seq approach used in this study–i.e., initially exploring different combinations of enzymes and size selections to evaluate the expected number and distribution of RAD fragments produced in the range of taxa of interest–demonstrates the importance of choosing appropriate parameters for library construction given specific project objectives and funding. Irrespective of the taxonomic group studied or the research questions of interest, we suggest that projects focusing on non-model taxa undertake an initial exploratory analysis like the one done here to determine conditions appropriate for targeting a desired number of loci while minimizing sequencing costs.

### Locus identification pipelines

As in other studies [49,98–100], we found that employing different pipelines for locus identification and SNP calling yielded somewhat different results, despite using many of the same parameters (e.g., applying the same quality filters to the input reads, selecting only those loci with a minimum sequencing depth of 6x, filtering out loci not present in at least four samples). We found that the iPYRAD *denovo+reference* pipeline typically identified a greater number of unique clusters per sample than the other methods tested, but the *denovo* pipeline identified a larger number of variable loci per sample than either of the other iPYRAD methods. Researchers need to critically evaluate alternative approaches and consider how different pipelines can affect the final genotype matrix produced.

Comparing the different iPYRAD methods, the *denovo+reference* pipeline is likely to identify more clusters than the other pipelines for several reasons. First, some RAD tags may fail to align to a reference genome either because homologous sequences are not present in that genome or because homologous loci from different samples are too divergent from one another to align. Some of these sequences may end up being clustered, separately, *de novo*. Second, when a reference genome is assembled, repetitive or duplicated regions are typically collapsed into single clusters [101]. As a consequence, what might appear as multiple unique clusters in a *denovo* clustering process may align to the same position in the reference genome, reducing the number of overall clusters identified. For a similar reason, we also expect to see fewer clusters recovered for the *denovo* pipeline versus the *denovo+reference* pipeline. That is, whereas some distinct clusters may be filtered from the *denovo* pipeline due to low coverage, they may nonetheless be recovered in the *denovo+reference* pipeline because several different low-coverage clusters all map to the same position in the reference genome.

Contrary to the pattern for clusters per sample, the number of putative loci per sample and the number of loci in the final genotype matrix were generally highest in the *denovo* pipeline followed by the *denovo+reference* and *reference* pipelines (Fig 4, Table 5). This pattern likely reflects the fact that in the first step of both reference-based iPYRAD pipelines, the BWA algorithm that is used to map the RAD tags against the *Callithrix jacchus* genome performs a local alignment, which attempts to align small regions within a read while masking the rest of the sequence when it is unable to align the read in its entirety. This process is fundamentally different from that used in the *denovo* analysis, where ≥85% of an entire read has to be similar for it to be clustered with other reads. Thus, local alignments can result in the successful mapping of widely diverged reads, which is desirable in studies of divergent taxa or taxa with dissimilar sequences at a given locus. Given that the percentage of the sequence that has to be similar for a read to be mapped either to a reference genome or clustered with other reads varies dramatically between the reference-based and *denovo* pipelines, it is unsurprising that the reference-based pipelines initially identify many more clusters. However, these clusters–which may include non-homologous sequences–are then more aggressively filtered out in subsequent pipeline steps (e.g., those that remove clusters containing poor alignments and those that remove clusters identified as potential paralogs because they would imply more than two unique alleles in an individual).

Also, it bears mentioning that for our reference-based analyses, we used a reference genome that was not equally related to all of the samples in our dataset–i.e., *Callithrix* is nested well within the platyrrhine radiation. We would expect that mapping to a reference genome that is not equally closely related to all of the taxa in a study could result in a reduced ability to detect homologous loci across samples. Indeed, we found that the *denovo* pipeline identified a somewhat higher number of variable loci than either of the reference genome-based pipelines for a diverse group of living primates for which reference genomes are not available. This was particularly important for the present study as we were interested in recovering thousands of loci, distributed throughout the genome, to elucidate evolutionary relationships at different time scales. However, it is important to recognize that our results do not imply that using a *denovo* clustering approach is always preferable. Mapping putative loci to a reference genome can be very informative when a genome that is equally related to all samples in a study is available or when information on gene position and/or function is important (e.g., for candidate gene, linkage, or association studies) [102–104].

### Phylogenetic inferences based on ddRAD-seq marker data

A critical analytical parameter that needs to be considered in RAD-seq based phylogenomics is the clustering threshold, which determines the minimum sequence similarity level needed to identify putatively orthologous loci. Both simulation-based and empirical studies have suggested that setting a very high threshold can result in oversplitting of putatively orthologous loci and the elimination of potentially informative variation [105,106]. Even though using a low similarity threshold for clustering may result in incorrectly classifying paralogous sequences as orthologous, oversplitting can be more detrimental to making correct phylogenetic inferences [105]. Simulation studies also suggest that lower thresholds for clustering should be used when studying deep phylogenetic splits [105,107]. We found that as the clustering threshold increased, the number of putative loci identified per sample increased, but the total number of variable loci shared across taxa in the final genotype matrix decreased. Thresholds higher than ~92% resulted in a lower recovery of loci across taxa, probably due to a failure to recognize alternate alleles at homologous loci across taxa.

Another important parameter to coXnsider when inferring phylogenetic relationships based on ddRAD-seq data is the minimum number of samples that must share a given consensus locus for inclusion in the final genotype matrix. That is, this minimum sample threshold determines the tolerance for missing data in the final dataset. Missing data could be the result of either mutations in restriction enzyme recognition sites in some taxa or to allelic dropout in certain samples. Setting the minimum sample threshold too high may bias phylogenetic inference, as rapidly evolving loci that are informative for resolving recently diverged taxa may be discarded [40,108]. A number of recent studies have found that setting lower minimum taxa thresholds produces more robust and better supported RAD-seq based phylogenies [37,40,41,105–108]. Even though we did not test the effect of varying the minimum sample threshold in our phylogenetic analysis, we were able to successfully reconstruct a well-supported phylogeny for platyrrhines when using the lowest minimum number of samples threshold possible in iPYRAD (N = 4 samples).

### New World primate phylogeny

Phylogenetic relationships among New World primates have been studied extensively from a molecular perspective over the past 20 years [52,53,55–59,61,97,109]. Most studies have yielded the same evolutionary relationships among the families Pitheciidae, Atelidae, and Cebidae as well as consistent branching patterns among the different genera within the first two of those families (but see[55,56]. Nevertheless, these studies left a number of other questions about New World monkey evolutionary relationships at the subfamily, genus, and species levels unresolved or poorly resolved, e.g., the arrangement of the three subfamilies (Aotinae, Callitrichinae, and Cebinae) within the Family Cebidae [55,57,61,110].

Our results based on ddRAD-seq marker data robustly reconstruct the same topology as seen in other molecular studies for the three main platyrrhine families, with all three clades being monophyletic and with the Family Pitheciidae as the basal within the radiation [52–54,57–59]. Additionally, our phylogeny elucidated some of the unresolved interspecific phylogenetic relationships among different species of robust capuchins (genus *Sapajus*). For example, while previous phylogenetic analyses using both nuclear and mitochondrial loci have clearly demonstrated the monophyly of each of the two capuchin genera–the “gracile” capuchins (genus *Cebus*) and the “robust” capuchins (genus *Sapajus*) [111,112]–these studies have not provided sufficient resolution to evaluate whether all of putative species of robust capuchin species represent monophyletic lineages. A recent study using sequence data from three mtDNA genes, found strong support only for monophyly of the species *Sapajus xanthosteros*, *S*. *nigritus*, and *S*. *robustus*, but all of the other *Sapajus* species fell into one large, widely distributed clade [113]. Our current analysis, by contrast, recovered strong support for reciprocal monophyly of both *S*. *libidinosus* and *S*. *flavius* within the robust capuchin radiation.

As noted above, inclusion of *Aotus* (owl monkeys) in some of our phylogenetic analyses resulted in some interesting uncertainties about the placement of this genus that speak to longstanding controversies in platyrrhine systematics. The position of *Aotus* within the New World monkey phylogeny has, historically, been contentious and confused. *Aotus* is remarkably convergent with titi monkeys (genera *Callicebus*, *Plecturocebus*, *and Cheracebus*, from the Family Pitheciidae) in a number of morphological and behavioral features (e.g., small body size, lack of sexual dimorphism in body and canine size, “socially-monogamous” or “pair-living” grouping patterns, heavy male investment in offspring care). However, prior molecular studies have consistently aligned *Aotus* with the cebids and not with the pitheciids, a result that is strongly confirmed in our study. Additionally, both our ML and coalescent-based analyses using the genotype matrix from the *denovo* pipeline provide marginally stronger support for a position for *Aotus* within the Cebidae as sister to the Callitrichinae (marmosets and tamarins), a position that has also been supported, albeit weakly, in other genome-wide studies that have utilized sequence data from multiple nuclear and mtDNA coding loci [58,59,97]. By contrast, our ML and coalescent-based analyses using the genotype matrices from the two reference-based pipelines provide marginally stronger support for a basal position of *Aotus* within the Cebidae.

Overall, our results provide comparable or better resolution to other molecular studies of platyrrhine phylogenetic history [52,55,58,97,114] and reiterate the challenge of pinpointing the phylogenetic placement of the genus *Aotus*. Our ambiguous results concerning the position of *Aotus*–which are based on a large number of presumably neutral SNP loci–are consistent with the different phylogenetic positions inferred for the genus based individual coding loci from both the nuclear and mitochondrial genomes [97,114] and suggest a rapid diversification among the early cebids marked by incomplete lineage sorting and perhaps significant gene flow or hybridization among incipient cebid lineages [17]. Incomplete lineage sorting is common in recently-diverged clades [115], but it can also occur in clades that have undergone early and rapid radiations [109, 100]. Additionally, it is notable that most genetic studies–ours included–have inferred that a large amount of evolutionary change occurred on the branch leading to crown *Aotus* from its common ancestor with other cebids, and long branches also lead to the crown nodes for other cebid (and other platyrrhine) genera, leading some researchers to argue that long branch attraction [116] may be contributing to poor resolution for the placement of *Aotus*. Future genomic research on New World monkeys should focus on exploring the early evolutionary history of the cebids and on disentangling whether and how incomplete lineage sorting and other factors such as introgression, hybridization, and long branch attraction complicate our assessment of this history.

## Conclusions

In the past, inferring the evolutionary relationships among extant New World monkey genera and species has proven difficult, in part because of the challenge of identifying markers capable of resolving relationships at both recent and deeper divergence dates. The evolutionary history of platyrrhines was characterized by an early, rapid diversification into three lineages corresponding to the three extant New World monkey families [52,57,117], with short phylogenetic branches between these clades that contain few diagnostic character states. Similarly, the more recent history of divergences among genera, species and subspecies within particular platyrrhine genera has also been difficult to resolve with confidence using a limited number of traditional sequence-based markers due to incomplete lineage sorting and, in some cases, hybridization.

Our results demonstrate the utility and promise of using a standard, cost-effective ddRAD-seq approach to identify large numbers of variable loci, evenly distributed across the genome, that can provide high phylogenetic resolution at a range of taxonomic levels and evolutionary time depths within a diverse and deep radiation of primates. Our study reveals an exciting future for primatology, as we successfully produced vast quantities of genome-wide data affordably and with relative ease. Moreover, analyses in progress of 78 samples from 10 different populations of one species included in this study (*Saguinus leucopus*) demonstrate that the same general ddRAD-seq protocol identified ~30,000 loci that are variable within this single species and are informative for studying population genetic structure over a fine geographic scale (Valencia et al., in preparation). As has been shown for other taxonomic groups [47,118–120], ddRAD-seq data should allow primatologists–and other biologists working with non-model taxa–to address a host of long standing questions that were previously difficult to tackle because of technological or financial constraints.

## Supporting information

S1 Fig Overview of the ddRAD-seq protocol followed in this study. (TIF)

S2 Fig Distribution of the number of heterozygous sites (Hs) and number of uncalled bases (Ns) in each cluster within each sample. Hs and Ns are calculated for all the reads that overlapped (merged) as well as for those R1 and R2 reads that did not overlap. 95% CI shown in black.
(TIF)

S3 Fig Distribution of the number of SNP sites present in the loci recovered across samples. The number of SNPs were calculated for all the reads that overlapped (merged) as well as for those R1 and R2 reads that did not overlap. 95% CI shown in black.
(TIF)

S4 Fig Number of reads and number of putative loci per sample across sample types. Hair samples have significantly fewer reads and consensus loci than blood or tissue samples.
(TIF)

S5 Fig Mapping loci discovered using the *denovo* pipeline to the chromosome 1 of the *Callithrix jacchus* reference genome. Only 1% of loci mapped to the same genome locations, indicating that the pipeline successfully filtered out duplicate and paralogous loci. The pullout shows a blowup of a portion of the data for Chromosome 1 (shaded region), where the spatial distribution of those loci that mapped uniquely to the reference genome at a median distance between loci of 29,249 bp.
(TIF)

S6 Fig **Phylogenetic relationships among the samples included in our study (without *Aotus*) based on maximum likelihood analyses of loci identified through the (a) *denovo*, (b) *denovo+reference*, and (c) *reference* pipelines in iPYRAD.** In each figure, the three platyrrhine families are indicated by background shading (green: Cebidae, red: Atelidae, blue: Pitheciidae). Numbers in each xf indicate nonparametric bootstrap support for the adjacent node. All unlabeled nodes had 100% bootstrap support. The position of Aotus is indicated in bold and by an arrow in each figure.
(TIF)

S7 Fig **Phylogenetic relationships among the samples included in our study based on quartet multispecies coalescent analyses of loci identified through the (a) *denovo*, (b) *denovo+reference*, and (c) *reference* pipelines in iPYRAD.** In each figure, the three platyrrhine families are indicated by background shading (green: Cebidae, red: Atelidae, blue: Pitheciidae). Numbers in each figure indicate nonparametric bootstrap support for the adjacent node. All unlabeled nodes had 100% bootstrap support. The position of Aotus is indicated in bold and by an arrow in each figure
(PDF)

S8 Fig Correlation between the number of loci shared among all of the samples within each clade of New World monkeys and clade divergence time estimated in fossil-calibrated molecular studies [52,53,58]. Irrespective of the divergence time estimates, as the genetic divergence between clades increases, the number of homologous loci shared across taxa decreases.
(TIF)

S1 Table Number of RAD tags recovered with each enzyme pair combination under different size selection windows. (DOCX)

S2 Table Number of total loci present in the final genotype matrix of 33 samples used for phylogenetic analyses and the number of those loci that are variable loci as the minimum number of samples in which a locus must be present for its inclusion in the data matrix decreases. (DOCX)

S3 Table Number of reads and number of putative loci recovered for each of the three replicates processed for four individual samples from across the platyrrhine radiation. We report the number of loci shared across all replicates as well as the percentage of loci for each replicate sample that were shared with other both other replicates.
(DOCX)

S1 File Laboratory protocol used in the University of Texas at Austin’s Genome Sequencing and Analysis Facility for to prepare ddRAD-seq libraries for next-generation sequencing on the Illumina HiSeq 2500 and Hi Seq 4000 platforms. (DOCX)
