# Supplementary figures and images for: A RAD-sequencing approach to genome-wide marker discovery, genotyping, and phylogenetic inference in a diverse radiation of primates
Source: PLoS One. 2018 Aug 17;13(8):e0201254. doi: 10.1371/journal.pone.0201254 (PMC6097672; doi:10.1371/journal.pone.0201254)

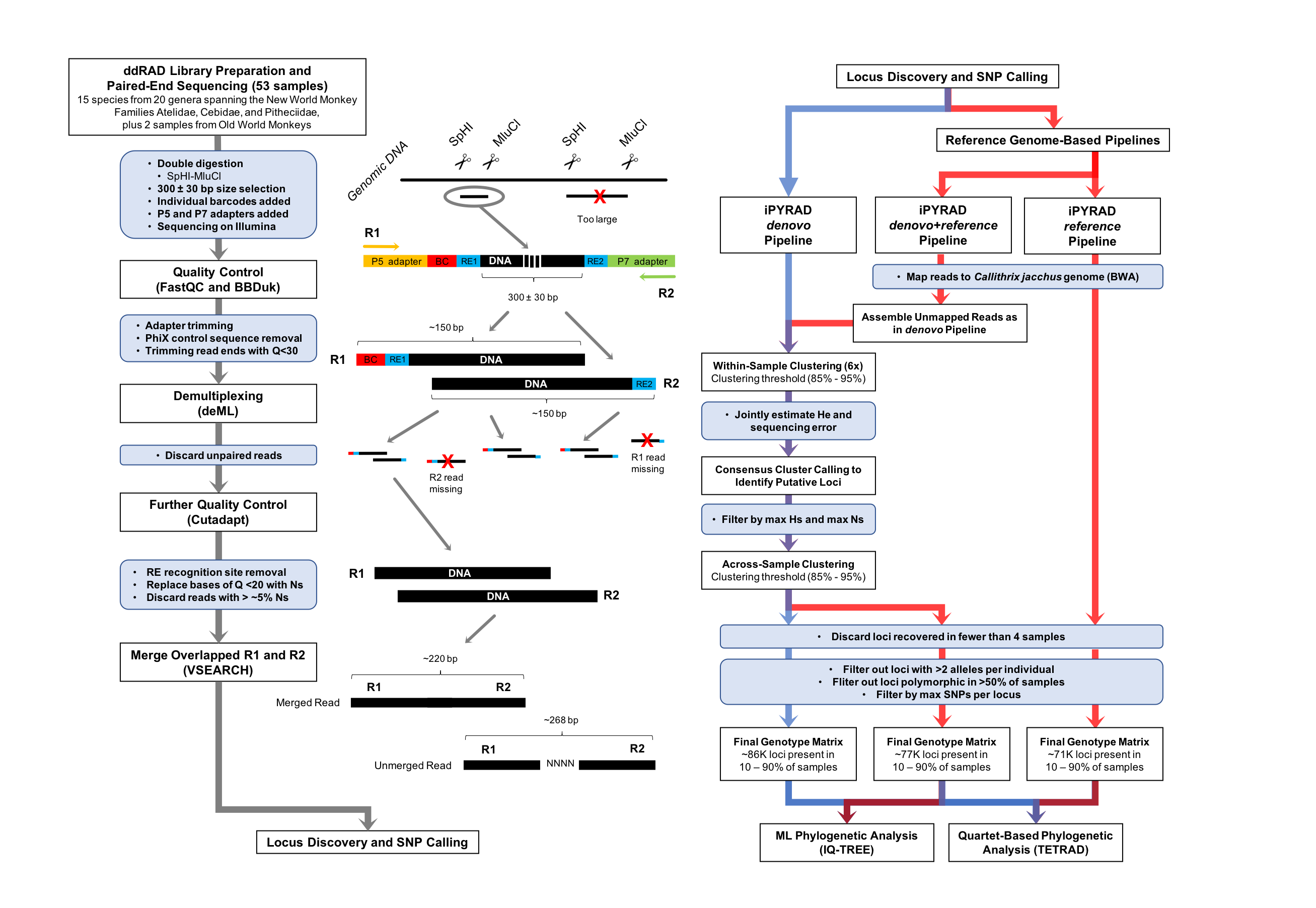

Supplement: S1 Fig — (TIF) [file pone.0201254.s001.tif]

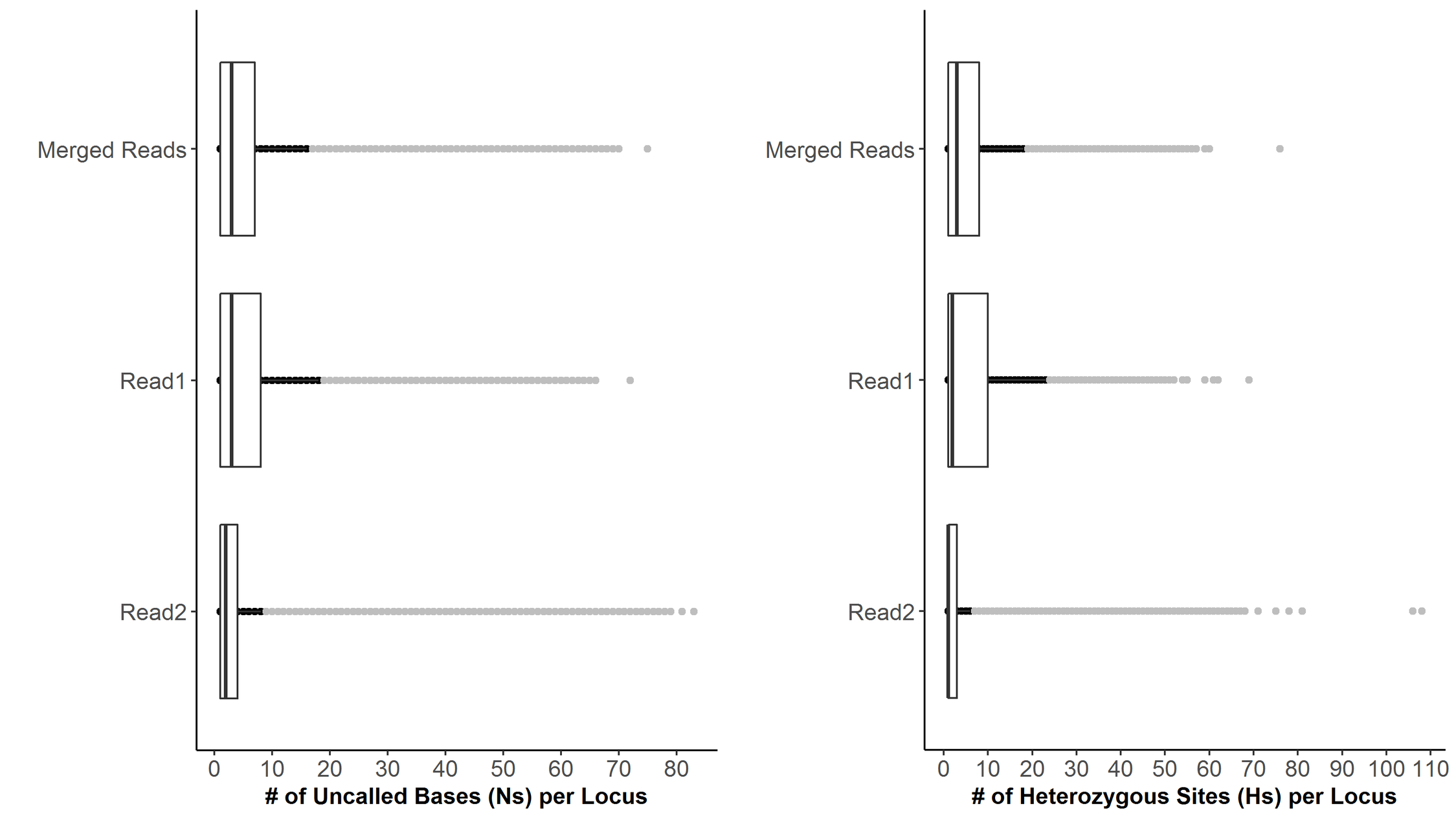

Supplement: S2 Fig — Hs and Ns are calculated for all the reads that overlapped (merged) as well as for those R1 and R2 reads that did not overlap. 95% CI shown in black. (TIF) [file pone.0201254.s002.tif]

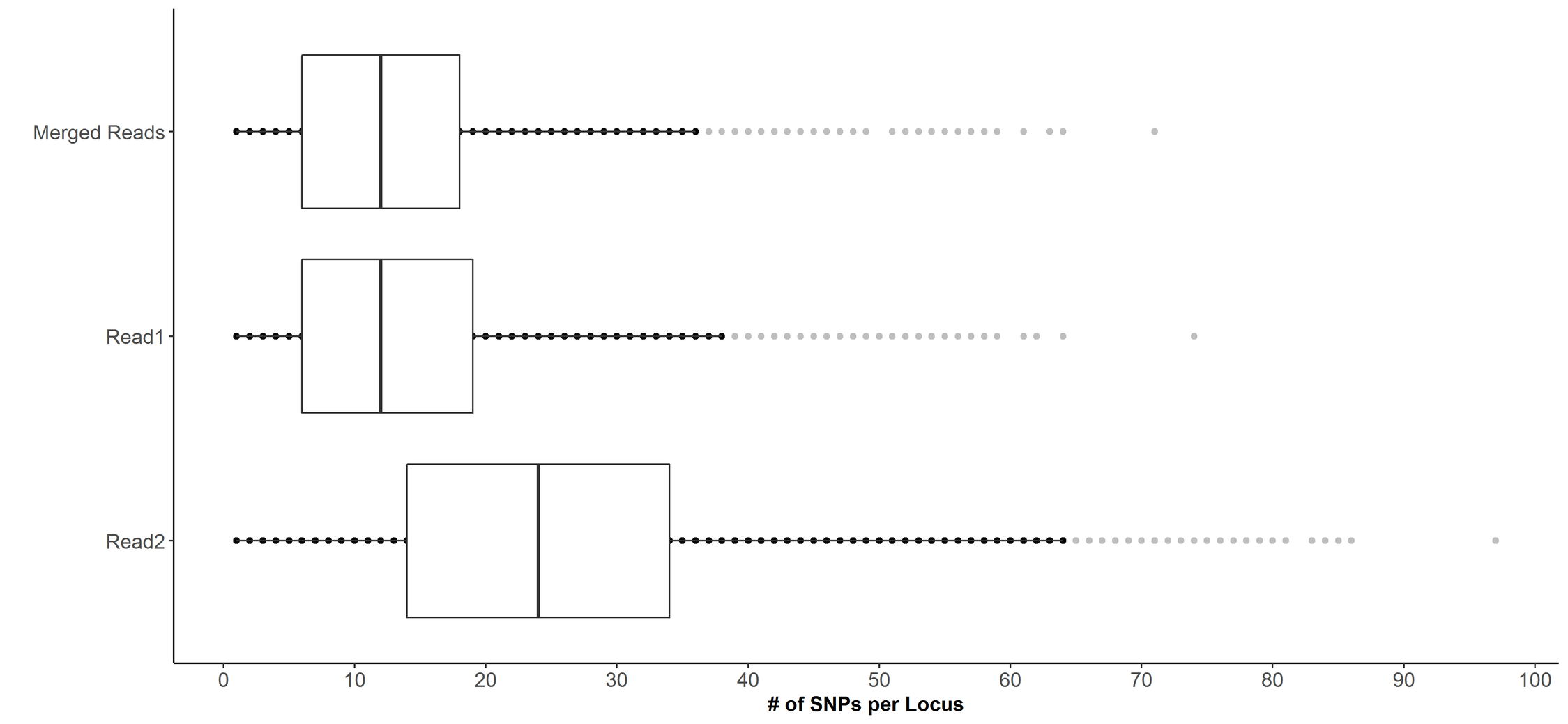

Supplement: S3 Fig — The number of SNPs were calculated for all the reads that overlapped (merged) as well as for those R1 and R2 reads that did not overlap. 95% CI shown in black. (TIF) [file pone.0201254.s003.tif]

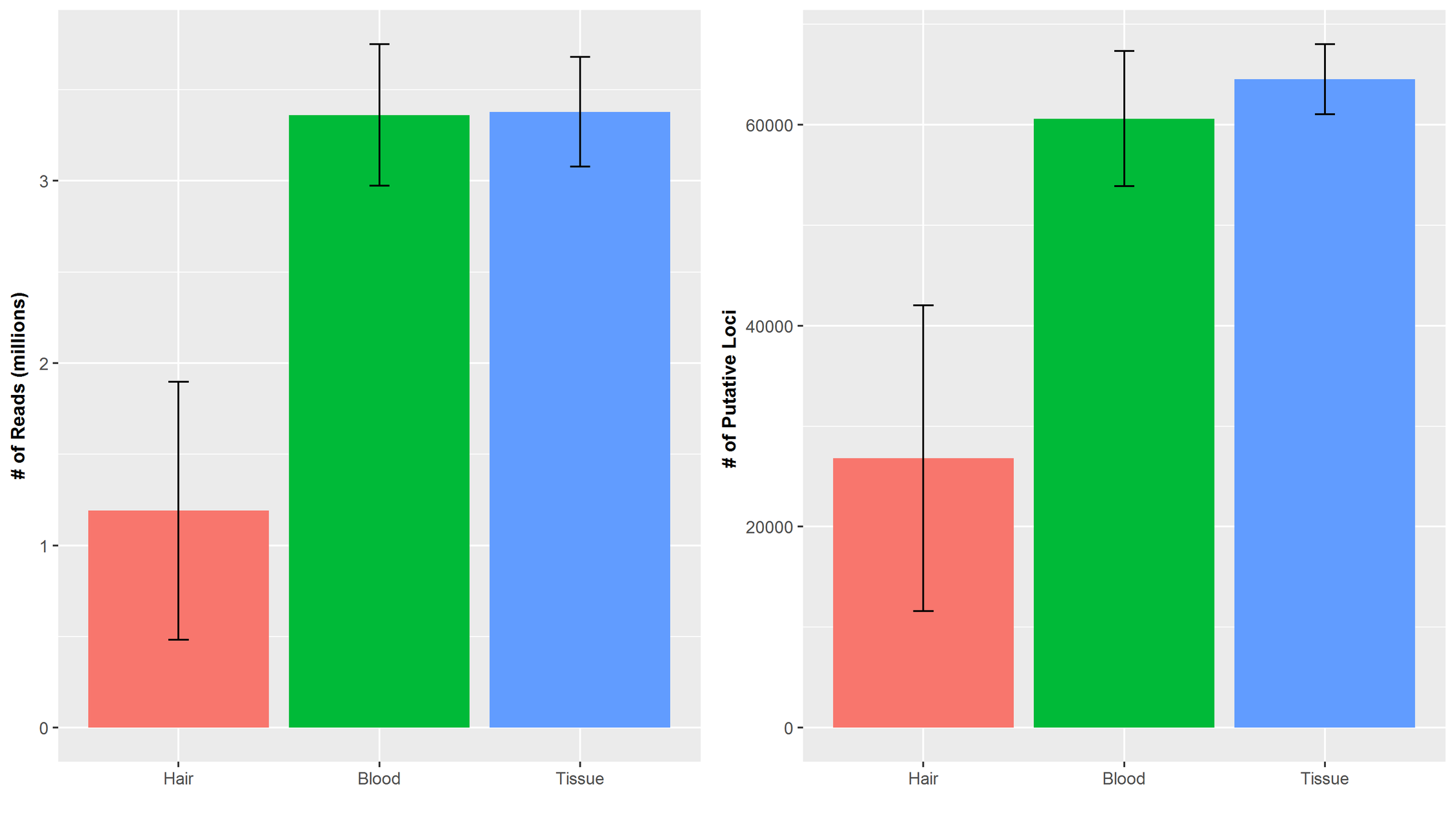

Supplement: S4 Fig — Hair samples have significantly fewer reads and consensus loci than blood or tissue samples. (TIF) [file pone.0201254.s004.tif]

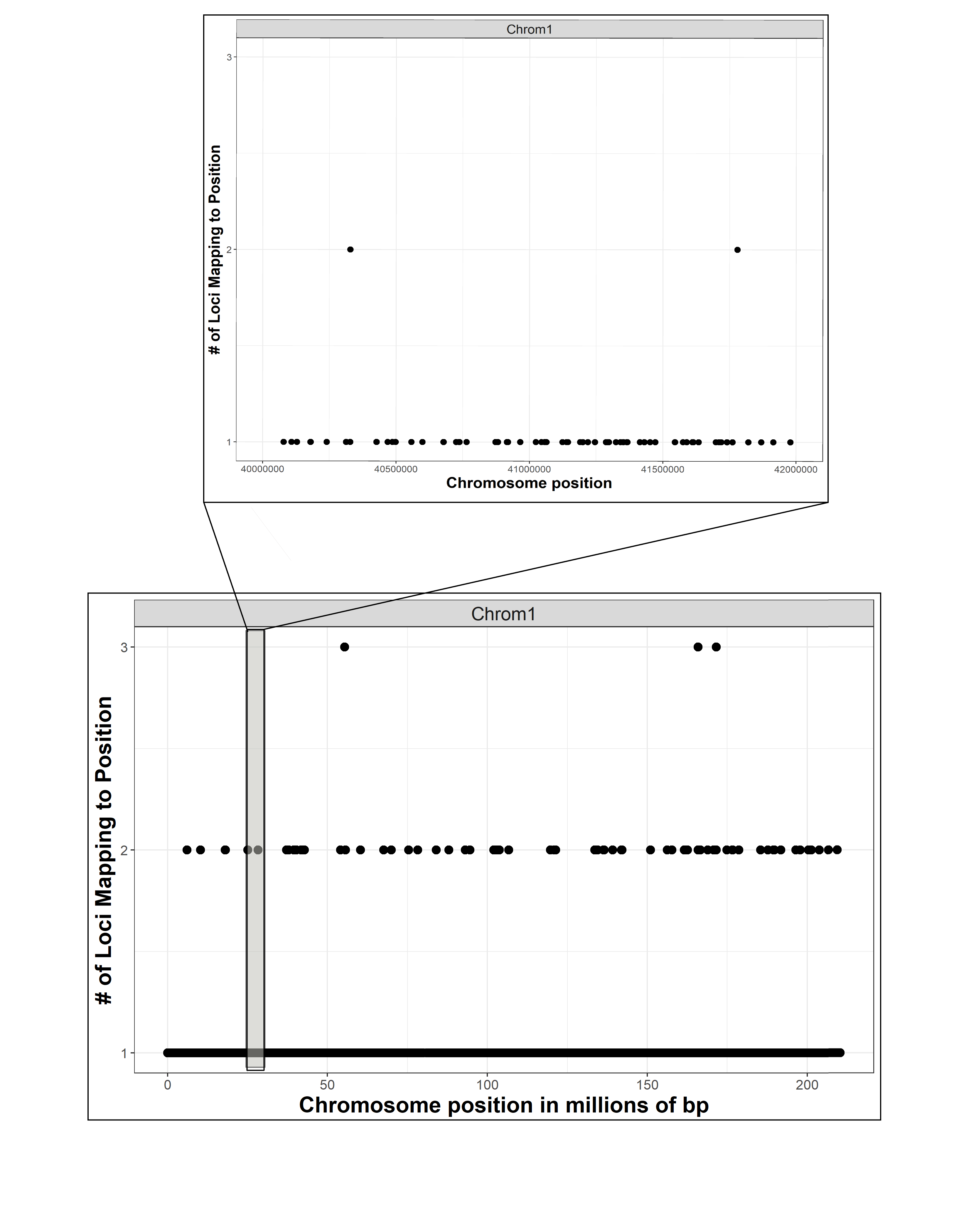

Supplement: S5 Fig — Only 1% of loci mapped to the same genome locations, indicating that the pipeline successfully filtered out duplicate and paralogous loci. The pullout shows a blowup of a portion of the data for Chromosome 1 (shaded region), where the spatial distribution of those loci that mapped uniquely to the reference genome at a median distance between loci of 29,249 bp. (TIF) [file pone.0201254.s005.tif]

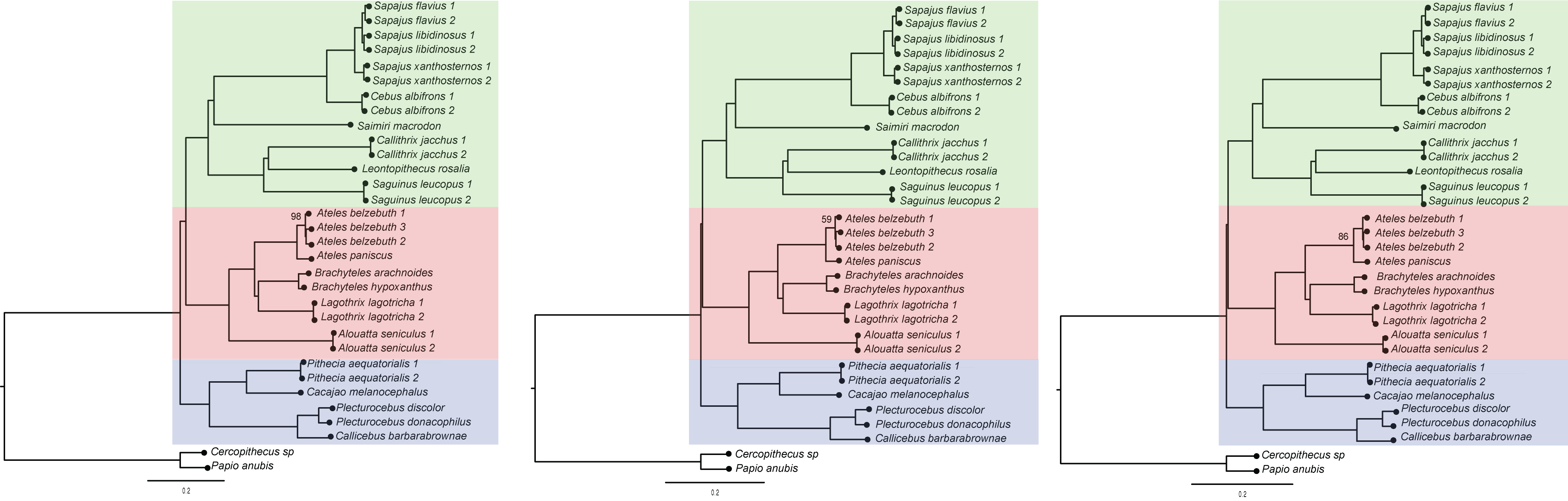

Supplement: S6 Fig — Phylogenetic relationships among the samples included in our study (without Aotus) based on maximum likelihood analyses of loci identified through the (a) denovo, (b) denovo+reference, and (c) reference pipelines in iPYRAD. In each figure, the three platyrrhine families are indicated by background shading (green: Cebidae, red: Atelidae, blue: Pitheciidae). Numbers in each xf indicate nonparametric bootstrap support for the adjacent node. All unlabeled nodes had 100% bootstrap support. The position of Aotus is indicated in bold and by an arrow in each figure. (TIF) [file pone.0201254.s006.tif]

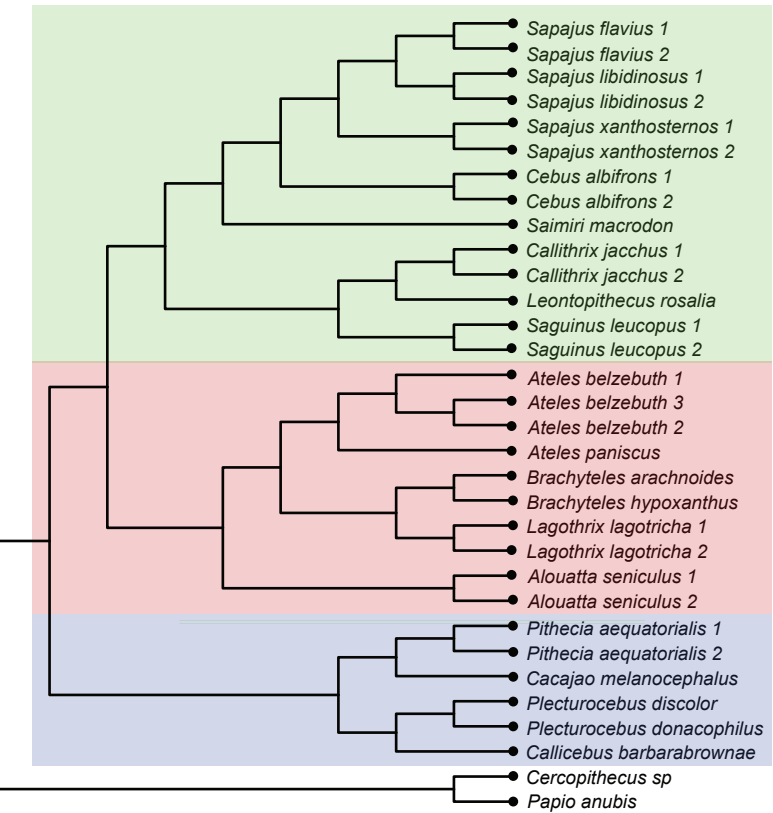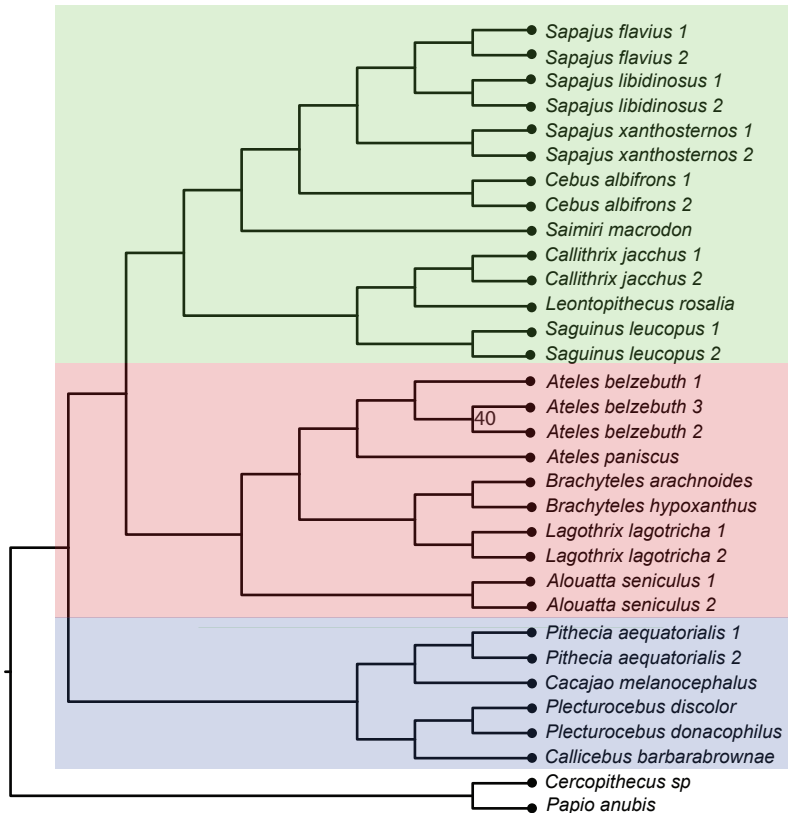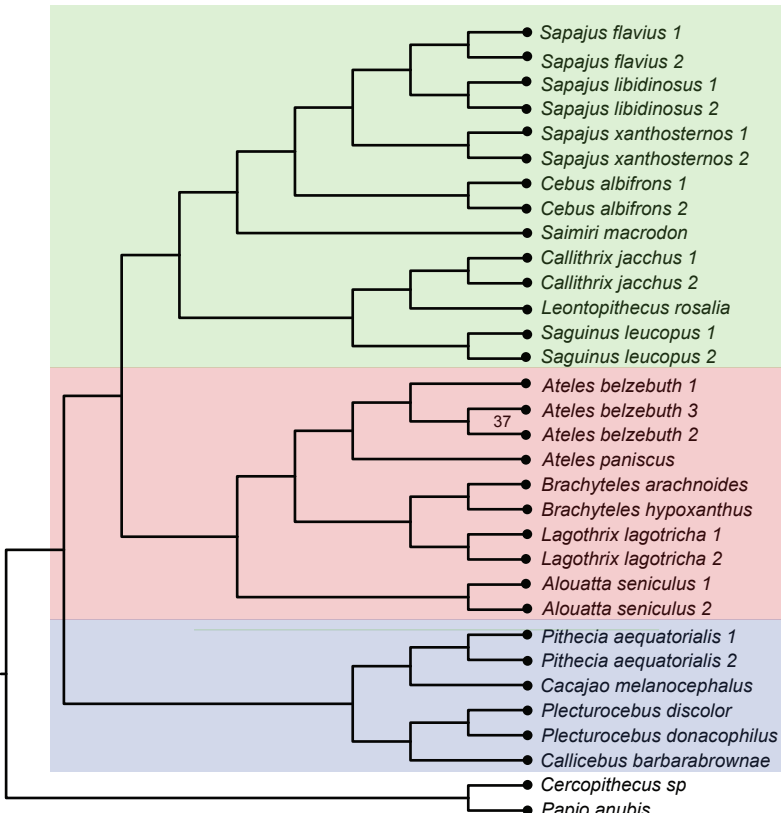

Supplement: S7 Fig — Phylogenetic relationships among the samples included in our study based on quartet multispecies coalescent analyses of loci identified through the (a) denovo, (b) denovo+reference, and (c) reference pipelines in iPYRAD. In each figure, the three platyrrhine families are indicated by background shading (green: Cebidae, red: Atelidae, blue: Pitheciidae). Numbers in each figure indicate nonparametric bootstrap support for the adjacent node. All unlabeled nodes had 100% bootstrap support. The position of Aotus is indicated in bold and by an arrow in each figure (PDF) [file pone.0201254.s007.pdf]

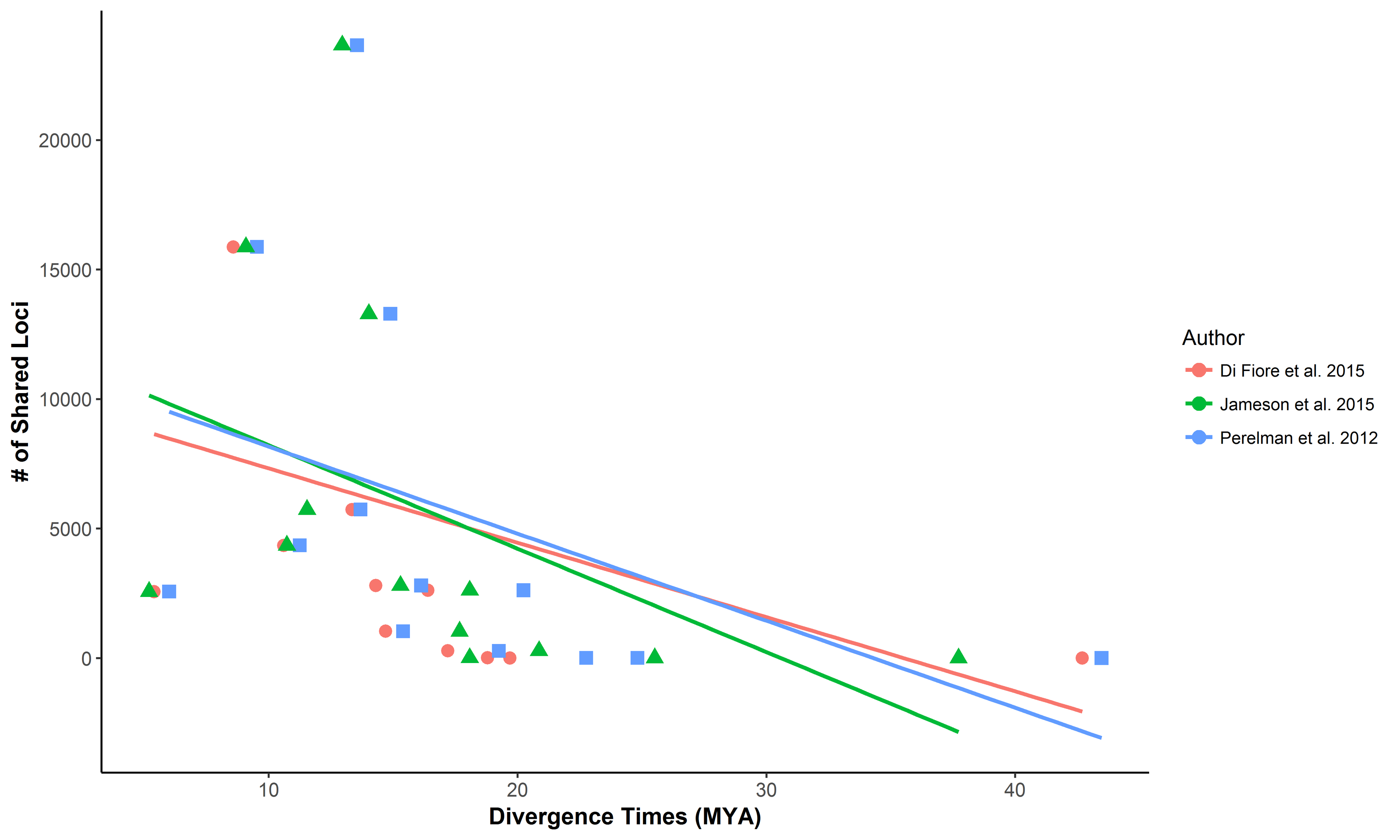

Supplement: S8 Fig — Irrespective of the divergence time estimates, as the genetic divergence between clades increases, the number of homologous loci shared across taxa decreases. (TIF) [file pone.0201254.s008.tif]
