# Supplementary material for: A RAD-sequencing approach to genome-wide marker discovery, genotyping, and phylogenetic inference in a diverse radiation of primates
Source: PLoS One. 2018 Aug 17;13(8):e0201254. doi: 10.1371/journal.pone.0201254 (PMC6097672; doi:10.1371/journal.pone.0201254)
Supplement: S1 Table — (DOCX) [file pone.0201254.s009.docx]

|  |  | Size Selection | | | | |
| --- | --- | --- | --- | --- | --- | --- |
| ENZYME PAIR | **Species** | **100 bp** | **200 bp** | **300 bp** | **400 bp** | **500 bp** |
| EcoRI-MspI | *Callicebus barbarabrownae* | 16061 | 14434 | 14828 | 19768 | 24298 |
|  | *Plecturocebus discolor* | 195319 | 220217 | 131239 | 85413 | 67562 |
|  | *Saguinus leucopus 1* | 68107 | 58813 | 38237 | 33661 | 29396 |
|  | *Saguinus leucopus 2* | 19185 | 46425 | 34253 | 32213 | 31147 |
|  | *Cebus albifrons* | 67198 | 57730 | 46717 | 37811 | 37362 |
|  | *Leontopithecus rosalia* | 231641 | 292968 | 218341 | 140494 | 10516 |
|  | *Pithecia aequatorialis* | 64209 | 100782 | 67212 | 49135 | 43511 |
|  | *Sapajus flavius* | 57613 | 66691 | 39661 | 33705 | 30694 |
| SpHI-MluCl | *Callicebus barbarabrownae* | 163762 | 84857 | 40932 | 18203 | 11325 |
|  | *Plecturocebus discolor* | 147379 | 84196 | 39823 | 17817 | 11099 |
|  | *Saguinus leucopus 1* | 128188 | 78297 | 42733 | 18655 | 11249 |
|  | *Saguinus leucopus 2* | 132904 | 82852 | 43653 | 19132 | 11183 |
|  | *Cebus albifrons* | 149152 | 78733 | 38553 | 17727 | 9769 |
|  | *Leontopithecus rosalia* | 146573 | 83020 | 42427 | 16938 | 9425 |
|  | *Pithecia aequatorialis* | 159868 | 86647 | 41337 | 18498 | 10440 |
|  | *Sapajus flavius* | 122734 | 75491 | 39056 | 18019 | 11528 |
| NlaIII-MluCl | *Callicebus barbarabrownae* | 1730663 | 733297 | 253454 | 91723 | 47292 |
|  | *Plecturocebus discolor* | 1809355 | 740031 | 249871 | 84547 | 41887 |
|  | *Saguinus leucopus 1* | 1977456 | 740674 | 235683 | 73807 | 31623 |
|  | *Saguinus leucopus 2* | 1996688 | 721756 | 231877 | 74761 | 33651 |
|  | *Cebus albifrons* | 2285222 | 591095 | 247324 | 65706 | 30787 |
|  | *Leontopithecus rosalia* | 1916759 | 807886 | 257044 | 82578 | 33592 |
|  | *Pithecia aequatorialis* | 1708174 | 696644 | 231367 | 83722 | 39175 |
|  | *Sapajus flavius* | 1853457 | 707802 | 261943 | 68307 | 29679 |
