# Supplementary material for: A RAD-sequencing approach to genome-wide marker discovery, genotyping, and phylogenetic inference in a diverse radiation of primates
Source: PLoS One. 2018 Aug 17;13(8):e0201254. doi: 10.1371/journal.pone.0201254 (PMC6097672; doi:10.1371/journal.pone.0201254)
Supplement: S2 Table — (DOCX) [file pone.0201254.s010.docx]

| Minimum # of Samples a Locus must be Present in to be Included in the Final Genotype Matrix | # of Samples | # of Loci in the Final Genotype Matrix | Total # of Variable Loci |
| --- | --- | --- | --- |
| 100% | 33 | 0 | 0 |
| 90% | 29 | 137 | 118 |
| 80% | 26 | 349 | 321 |
| 70% | 23 | 647 | 612 |
| 60% | 19 | 1,570 | 1,531 |
| 50% | 16 | 3,163 | 3,124 |
| 40% | 13 | 6,629 | 6,590 |
| 30% | 9 | 19,049 | 19,006 |
| 20% | 6 | 48,089 | 47,797 |
| 12% | 4 | 86,407 | 84,834 |
