# Supplementary material for: A RAD-sequencing approach to genome-wide marker discovery, genotyping, and phylogenetic inference in a diverse radiation of primates
Source: PLoS One. 2018 Aug 17;13(8):e0201254. doi: 10.1371/journal.pone.0201254 (PMC6097672; doi:10.1371/journal.pone.0201254)
Supplement: S3 Table — We report the number of loci shared across all replicates as well as the percentage of loci for each replicate sample that were shared with other both other replicates. (DOCX) [file pone.0201254.s011.docx]

| Sample | # of Reads | # of Putative Loci | # of Loci Shared Across all Replicates | % of Putative Loci Shared with Other Replicates |
| --- | --- | --- | --- | --- |
| *Callicebus barbarabrownae* | | | | |
| Replicate 1 | 3324174 | 73116 | 39702 | 54.3 |
| Replicate 2 | 4054887 | 76604 |  | 51.8 |
| Replicate 3 | 1066732 | 50637 |  | 78.4 |
| Average |  |  |  | **61.5** |
|  | | | | |
| *Plecturocebus discolor* | | | | |
| Replicate 1 | 2757348 | 66302 | 42027 | 63.4 |
| Replicate 2 | 4470227 | 68952 |  | 61.0 |
| Replicate 3 | 2266428 | 64637 |  | 65.0 |
| Average |  | | | **63.1** |
|  | | | | |
| *Saguinus leucopus* | | | | |
| Replicate 1 | 2414452 | 67783 | 43133 | 63.6 |
| Replicate 2 | 2468246 | 61898 |  | 69.7 |
| Replicate 3 | 1729963 | 61151 |  | 70.5 |
| Average |  | | | **67.9** |
|  | | | | |
| *Ateles belzebuth* | | | | |
| Replicate 1 | 2482530 | 62777 | 43246 | 68.9 |
| Replicate 2 | 2472530 | 62452 |  | 69.2 |
| Replicate 3 | 5055832 | 77799 |  | 55.6 |
| Average |  | | | **68.9** |
