# Supplementary material for: A RAD-sequencing approach to genome-wide marker discovery, genotyping, and phylogenetic inference in a diverse radiation of primates
Source: PLoS One. 2018 Aug 17;13(8):e0201254. doi: 10.1371/journal.pone.0201254 (PMC6097672; doi:10.1371/journal.pone.0201254)
Supplement: S1 File — (DOCX) [file pone.0201254.s012.docx]

**Double-digest Restriction Association DNA (ddRAD) Sequencing Protocol**

**Genomic Analysis and Sequencing Facility – University of Texas at Austin**

**Procedure**

1. **Double digestion of gDNAs**
   1. Add 10ul of 10ng/ul gDNA sample into each well of a 96 well plate. (If less than 10 µl of gDNA is used, add the appropriate volume of nuclease-free water to bring samples up to 10 µl volume.)
   2. Prepare samples for double digestion by creating the following Master Mix

| Component | Volume (µL) |
| --- | --- |
| Enzyme 1 (use 1 unit of RE per 100ng @ 10,000X) | 0.1 |
| Enzyme 2 (use 1 unit of RE per 100ng@ 10,000X) | 0.1 |
| Cutsmart Buffer | 3.0 |
| Nuclease-Free Water | 16.8 |
| *Total* | *20.0* |

- 1. Add 20 μL of master mix to the 96 well PCR plate containing 10 µl of each sample to be digested and incubate at 37°C for 2 hours. Cover plate with a foil seal.

1. **Sample purification using AMPure Bead XP purification**
   1. Let beads come to room temperature, then vortex thoroughly to resuspend.
   2. Spin the plate down and carefully remove the foil seal from the plate.
   3. Add 1.8X beads (54 μL/sample), creating a homogenous mixture. Cover with a foil seal, vortex gently, and spin briefly to collect the samples.
   4. Incubate mixture at room temperature for 15 minutes.
   5. Place the plate in the DynaMag magnetic plate rack until supernatant is clear (approximately 5 minutes).
   6. Remove the foil seal and carefully remove, then discard the supernatant ensuring no beads are discarded.
   7. Keeping the plate on the magnet, add 100 μL freshly prepared 80% ethanol, wait 30 seconds, and discard ethanol. Repeat.
   8. Using clean 20ul pipette tips, draw out any remaining 80% ethanol at the bottom of the wells.
   9. Air-dry beads at room temperature for 5 minutes on the magnet with the lid off. Do not over-dry the beads as this may result in significant yield loss.
   10. Remove the plate from the magnet and resuspend the beads in 22 μL nuclease-free water, pipet up and down to mix thoroughly, and incubate at room temperature for 2 minutes.
   11. Place the plate on a magnet until the supernatant is clear (approximately 5 minutes) and carefully transfer 20 μL to a clean PCR plate; do not transfer any beads.
2. **Adaptor ligation**
   1. Take 10 µl of each digested sample into adaptor ligation.
   2. Prepare samples for adaptor ligation by creating the following reaction**:**

| Component | Volume (µL) |
| --- | --- |
| P5 adaptor (4 µM) (Flex P5) | 2.0 |
| P7 adaptor (4 µM) (Flex P7) | 2.0 |
| Digested DNA | 10.0 |
| *Total* | *14.0* |

- 1. Separately, prepare the following ligation Master Mix:

| Component | Volume (µL) |
| --- | --- |
| T4 DNA Ligase | 1.0 |
| T4 DNA Ligase Buffer with 10mm ATP | 4.0 |
| Nuclease-Free | 21.0 |
| *Total* | *26.0* |

- 1. Combine the digested DNA and adaptor mix with 26 µl of master mix per reaction for a total of 40 µl per reaction. Cover plate with a foil seal. Mix by gently vortexing and spin down.
  2. Incubate at room temperature on bench top for 30 minutes.

1. **AMPure Double Bead clean-up to enrich for desired sized DNA fragments before pooling**
   1. Remove the AMPure XP beads from the refrigerator and allow the beads to warm to room temperature.
   2. Add 0.55x volume of AMPure beads to the DNA sample(s). Mix until homogenous by pipetting or vortexing gently.
   3. Incubate 10 minutes at room temperature.
   4. Collect beads on a magnet.
   5. Remove and save the supernatant in a new 96-well plate (this supernatant is enriched for fragments less than 700-800bp).
   6. Calculate the amount of beads to add to the transferred supernatant from step 4.5. Since the supernatant from Step 4.5 is already in the AMPure bead buffer, which contains PEG, you must reduce the amount of AMPure reagent added at this step. The relative amount of AMPure beads needed at this step can be calculated by subtracting the ratio from step 4.2 from the desired ratio for the lower limit (0.8x AMPure ratio for a lower size limit desired of ~100-150bp):

Example: 0.8 - 0.55 = 0.25.

Then, the volume of the supernatant in step 4.5 can be multiplied to this number to obtain the volume of beads needed.

In this example: 0.25 x 58 µl = 14.5 µl AMPure beads**.**

- 1. Add the calculated volume of beads to the supernatant from step 4.5 and mix until homogeneous by pipetting or vortexing gently.
  2. Incubate for 10 minutes at room temperature.
  3. Collect beads using the DynaMag 96-side magnet
  4. Carefully remove the supernant while avoiding any beads.
  5. Wash 2x with 100 µl freshly made 80% EtOH.
  6. Dry beads for 5 minutes at room temperature. Do not overdry the beads or the DNA will fail to elute efficiently.
  7. To elute the DNA from the beads, add 17 **µ**l of nuclease-free water, and resuspend the beads by pipetting up and down 10 times. Incubate for at room temperature for 2 minutes. Place the plate on the magnet to collect the beads (~5 min). Carefully remove 15 µl to a new 96 well plate. Avoid transferring any beads.
  8. Save 1.5 µl aliquot for troubleshooting.

1. **Pooling samples**
   1. Quantify the amount of DNA in each sample using the Pico Green Plate Based Assay
   2. Combine equal amounts of the ligated DNA that has been enriched for desired sizes in step 4 each sample to create pool(s). The GSAF has 24 different internal barcodes; if there are more than 24 samples, more than one pool will be created.
2. **Size Selection with Pippin Prep**
   1. Follow Pippin Prep protocol and size select for appropriate size based on customer. Use 1.5% blue Pippin gel with R2 marker.
   2. Purify using a MinElute column and elute 75 μL sterile water (37.5 µl x 2).
3. **PCR amplification of size selected DNA**
   1. Samples are run in triplicate.
   2. Prepare samples for PCR by creating the following PCR Master Mix per reaction:

| Component | Volume (µL) |
| --- | --- |
| DNA | 23.0 |
| 25 μM i5 | 1.0 |
| 25 μM i7 Primer | 1.0 |
| NEB Next 2x Hi-Fidelity Master Mix | 25.0 |
| *Total* | *50.0* |

- 1. Run in a thermocycler under the following conditions:

| Cycle Step | Temp | Time | # of Cycles |
| --- | --- | --- | --- |
| Initial Denaturation | 98 | 30 seconds | 1 |
| Denaturation | 98 | 10 seconds | 12 |
| Annealing | 65 | 30 seconds |  |
| Extension | 72 | 30 seconds |  |
| Final Extension | 72 | 5 minutes | 1 |
| Hold | 4 | Hold | 1 |

1. **Purify the samples using AMPure Bead XP purification**
   1. Let beads come to room temperature, then vortex thoroughly to resuspend.
   2. Combine the 3 PCR reactions into a 1.5 ml tube for AMPure bead clean up.
   3. Add 0.8X beads (120 µL/sample), using a multichannel pipettor, pipet up and down to create a homogenous mixture.
   4. Incubate mixture on bench for 15 minutes at room temperature.
   5. Place the tubes in a magnetic plate rack until supernatant is clear (approximately 5 minutes).
   6. Carefully remove and discard the supernatant, ensuring no beads are discarded.
   7. Prepare fresh 80% ethanol.
   8. Keeping the tubes on the magnet, add 300 µL freshly prepared 80% ethanol, wait 30 seconds, and discard ethanol.
   9. Repeat step 10.8 once.
   10. Use fresh pipette tips and draw out any remaining 80% ethanol at the bottom of the wells.
   11. Air-dry beads at room temperature for 5-10 minutes on the magnet with the lid off. Caution: over-drying the beads may result in dramatic yield loss.
   12. Remove the plate from the magnet.
   13. Resuspend the beads in 32 μL nuclease-free water, pipet up and down to mix thoroughly, and incubate at room temperature for 2 minutes.
   14. Place the plate on a magnet until the supernatant is clear (approximately 5 minutes).
   15. Carefully transfer 30 μL to a clean 1.5mL Eppendorf tube, making sure not to transfer any beads. This is the DNA to be sequenced.
